# Pharmacological characterisation of novel adenosine A_3_ receptor antagonists

**DOI:** 10.1038/s41598-020-74521-y

**Published:** 2020-11-27

**Authors:** Kerry Barkan, Panagiotis Lagarias, Margarita Stampelou, Dimitrios Stamatis, Sam Hoare, Dewi Safitri, Karl-Norbert Klotz, Eleni Vrontaki, Antonios Kolocouris, Graham Ladds

**Affiliations:** 1 Department of Pharmacology, University of Cambridge, Tennis Court Road, Cambridge, CB2 1PD UK; 2 Section of Pharmaceutical Chemistry, Department of Pharmacy, School of Health Sciences, National and Kapodistrian University of Athens, Panepistimiopolis-Zografou, 15771 Athens, Greece; 3 Pharmechanics LLC, 14 Sunnyside Drive South, Owego, NY 13827 USA; 4 Pharmacology and Clinical Pharmacy Research Group, School of Pharmacy, Bandung Institute of Technology, Bandung, 40534 Indonesia; 5 Institute of Pharmacology and Toxicology, University of Würzburg, Versbacher Str. 9, 97078 Würzburg, Germany

**Keywords:** Receptor pharmacology, Molecular modelling, Extracellular signalling molecules

## Abstract

The adenosine A_3_ receptor (A_3_R) belongs to a family of four adenosine receptor (AR) subtypes which all play distinct roles throughout the body. A_3_R antagonists have been described as potential treatments for numerous diseases including asthma. Given the similarity between (adenosine receptors) orthosteric binding sites, obtaining highly selective antagonists is a challenging but critical task. Here we screen 39 potential A_3_R, antagonists using agonist-induced inhibition of cAMP. Positive hits were assessed for AR subtype selectivity through cAMP accumulation assays. The antagonist affinity was determined using Schild analysis (pA_2_ values) and fluorescent ligand binding. Structure–activity relationship investigations revealed that loss of the 3-(dichlorophenyl)-isoxazolyl moiety or the aromatic nitrogen heterocycle with nitrogen at α-position to the carbon of carboximidamide group significantly attenuated K18 antagonistic potency. Mutagenic studies supported by molecular dynamic simulations combined with Molecular Mechanics—Poisson Boltzmann Surface Area calculations identified the residues important for binding in the A_3_R orthosteric site. We demonstrate that K18, which contains a 3-(dichlorophenyl)-isoxazole group connected through carbonyloxycarboximidamide fragment with a 1,3-thiazole ring, is a specific A_3_R (< 1 µM) competitive antagonist. Finally, we introduce a model that enables estimates of the equilibrium binding affinity for rapidly disassociating compounds from real-time fluorescent ligand-binding studies. These results demonstrate the pharmacological characterisation of a selective competitive A_3_R antagonist and the description of its orthosteric binding mode. Our findings may provide new insights for drug discovery.

## Introduction

The adenosine A_3_ receptor (A_3_R), belongs to a family of four adenosine receptor (AR) subtypes (A_1_R, A_2A_R, A_2B_R and A_3_R), and is involved in a range of pathologies including cardiovascular, neurological and tumour-related diseases. Unsurprisingly therefore, A_3_R is a pharmaceutical target. Interestingly, the A_3_R has been described as enigmatic, whereby many of the effects attributed to A_3_Rs are contradictory^1^. Despite this, A_3_R antagonists having been described as potential treatments of asthma, chronic obstructive pulmonary disease (COPD) and glaucoma^2,3^, continuous research into antagonists at the A_3_R are warranted. It has also been suggested that the A_3_R is over expressed in various tumour cells suggesting it may be a viable drug target against cancer proliferation^4–6^. While a number of novel potent and selective A_3_R antagonists have been previously described^7–9^, one of the challenges associated with the druggability of the AR family has been the targeting of individual subtypes with sufficient specificity to limit off-target side effects^10^.

Although all AR members are activated by the endogenous agonist adenosine, the A_2A_R and A_2B_R are predominantly G_s_-coupled whereas A_1_R and A_3_R generally couple to G_i/o_. This classical pathway following A_3_R activation and G_i/o_ coupling is the inhibition of adenylate cyclase (AC) results in a decrease in cAMP levels, although extracellular signal-regulated kinase 1/2 (ERK1/2) activation has also been described^11^.

The A_1_R and A_2A_R are two of the best structurally characterised G protein-coupled receptors (GPCRs), with multiple structures available for both^12–16^, although the A_3_R structure is yet to be resolved. The limited availability of diverse high-resolution structures of the A_3_R bound to pharmacologically distinct ligands has meant there is a discrepancy between the capability to predict compound binding versus pharmacological behaviour^17^. In silico screening of vast compound libraries against receptor structures, known as structural-based drug design, offers huge potential in the development of highly selective ligands^18^. With this in mind, compounds K1-K25, K28 and K35, previously discovered as A_3_R ligands^19^, and the newly identified compounds K26, K27, K29-K34 and K36-K39 were pharmacologically characterised as potential antagonists using A_3_R-mediated inhibition of cAMP accumulation. Here, we describe the identification of a potent and selective A_3_R antagonist, K18 (O4-{[3-(2,6-dichlorophenyl)-5-methylisoxazol-4-yl]carbonyl}-2-methyl-1,3-thiazole-4-carbohydroximamide). Using molecular dynamic (MD) simulations, Molecular Mechanics—Poisson Boltzmann Surface Area (MM-PBSA) calculations and site-directed mutagenesis, we eluded its potential binding site. Kinetic binding experiments of K18 and its congener molecules K5, and K17 using a bioluminescence resonance energy transfer (BRET) method combined with functional assays led to the identification of important structural features of K18 for binding and activity. Further evaluation of this compound (and structurally related synthetic analogues) may afford a novel therapeutic benefit in pathologies such as inflammation and asthma.

## Results

### Identification of A_3_R selective antagonists

We initially conducted a blinded screen of 39 compounds (K1–39) to identify selective A_3_R antagonists some of which have previously been identified to bind A_1_R, A_3_R or A_2A_R using radio-labelled assays^19^. Our screen was carried out using A_3_R expressing Flp-In™-Chinese hamster ovary (CHO) cells where adenosine-3',5' cyclic monophosphate (cAMP) accumulation was detected following a combined stimulation of 10 μM forskolin (to allow A_3_R mediated G_i/o_ response to be observed), 1 μM tested compound and the predetermined IC_80_ concentration of 2S,3S,4R,5R)-5-(6-aminopurin-9-yl)-N-ethyl-3,4-dihydroxyoxolane-2-carboxamide (NECA) (3.16 nM). Compound K1-39 were identified by unblinding (Table 1 and Supplementary Table 1) and are hereinafter referred to as their denoted ‘K’ number. For the purpose of structure–activity relationships studies, 12 new compounds (K26, K27, K29–K34 and K36–K39) were assayed both functionally and through radioligand binding (Supplementary Table 1).

**Table 1:** Mean cAMP accumulation as measured in Flp-In CHO cells stably expressing A3R following stimulation with 10 μM forskolin only (DMSO) or 10 μM forskolin, NECA at the predetermined IC80 concentration and 1 μM test compound/MRS 1220/DMSO control.

Compound	Compound name	Chemical structure	cAMP accumulation		Radioligand binding (Ki (μM))^c^		
			Mean^a^	Mean difference^b^	A_3_R	A_1_R	A_2A_R
	NECA		60.32 ± 3.41	–	ND	ND	ND
	DMSO	CH_3_–SO–CH_3_	100.00 ± 1.15	− 39.68	ND	ND	ND
	MRS 1220		111.30 ± 1.65	− 50.95	ND	ND	ND
K1	HTS12884SC^1^		84.81 ± 4.90	− 24.49	**3.10**	> 100	**2.67**
K10	STK300529^1^		84.91 ± 5.37	− 24.59	**4.49**	> 60	> 60
K11	SKT323144^1^		80.78 ± 4.77	− 20.46	**5.15**	> 60	30
K17	SPB02734^1^		82.41 ± 7.55	− 22.09	**4.16**	> 30	> 60
K18	SPB02735^1^		102.6 ± 2.13	− 42.27	**0.89**	> 100	> 100
K20	GK03725^1^		97.86 ± 2.60	− 37.54	**0.91**	**1.09**	**7.29**
K23	GK01176^1^		88.02 ± 1.70	− 27.70	**1.65**	**1.18**	**4.69**
K25	GK01513^1^		88.66 ± 5.36	− 28.34	**1.55**	> 100	> 100
K32	STK323544		83.39 ± 5.27	− 23.07	**2.40**	> 100	> 100

Binding affinities were obtained through radioligand binding assays against the A_1_R, A_2A_R and A_3_R.

^a^cAMP accumulation mean ± SEM expressed as %10 μM forskolin response where *n* = 3 independent experimental repeats, conducted in duplicate. Potential antagonists were selected for further investigation based on a high mean cAMP accumulation (> 80%).

^b^Difference between the mean cAMP accumulation between ‘NECA’ and each compound expressed as %10 μM forskolin response.

^c^Binding affinity measured in three independent experiments and where indicated, previously published in Lagarias et al*.*^19^. Bold denotes binding affinity < 10 μM.

^1^Indicates previously published in Lagarias et al*.*^19^.

Co-stimulation with 10 μM of both forskolin and NECA reduced the cAMP accumulation when compared to 10 μM forskolin alone and this was reversed with the known A_3_R antagonist MRS 1220 (Table 1 and Supplementary Fig. 1). Compounds K1, K10, K11, K17, K18, K20, K23, K25 and K32 were identified as potential antagonists at the A_3_R through their ability to elevate cAMP accumulation when compared to forskolin and NECA co-stimulation. Of the nine potential A_3_R antagonists, eight (excluding K11) appeared to be antagonists at the tested concentration of 1 μM (Supplementary Fig. 2 and Supplementary Table 2).

A number of compounds previously documented (K5, K9, K21, K22 and K24;^19^ or determined in this study (K26, K27 and K34) to have sub-micromolar binding affinities for A_3_R showed no activity in our cAMP-based screen (Table 1, Supplementary Table 1). To ensure robustness of our functional screen, full inhibition curves of NECA in the presence or absence of tested compounds (1 μM or 10 μM) were constructed in A_3_R Flp-In CHO cells (Supplementary Fig. 3, Supplementary Table 3). In this preliminary data all nine compounds (K5, K9, K11, K21, K22, K24, K26, K27 and K34) appeared to reduce the NECA potency at the highest tested concentration (10 μM) but showed no effect at 1 μM and thus appear to be low potency antagonists at the A_3_R.

### AR subtype selectivity and specificity

**Figure 1:**
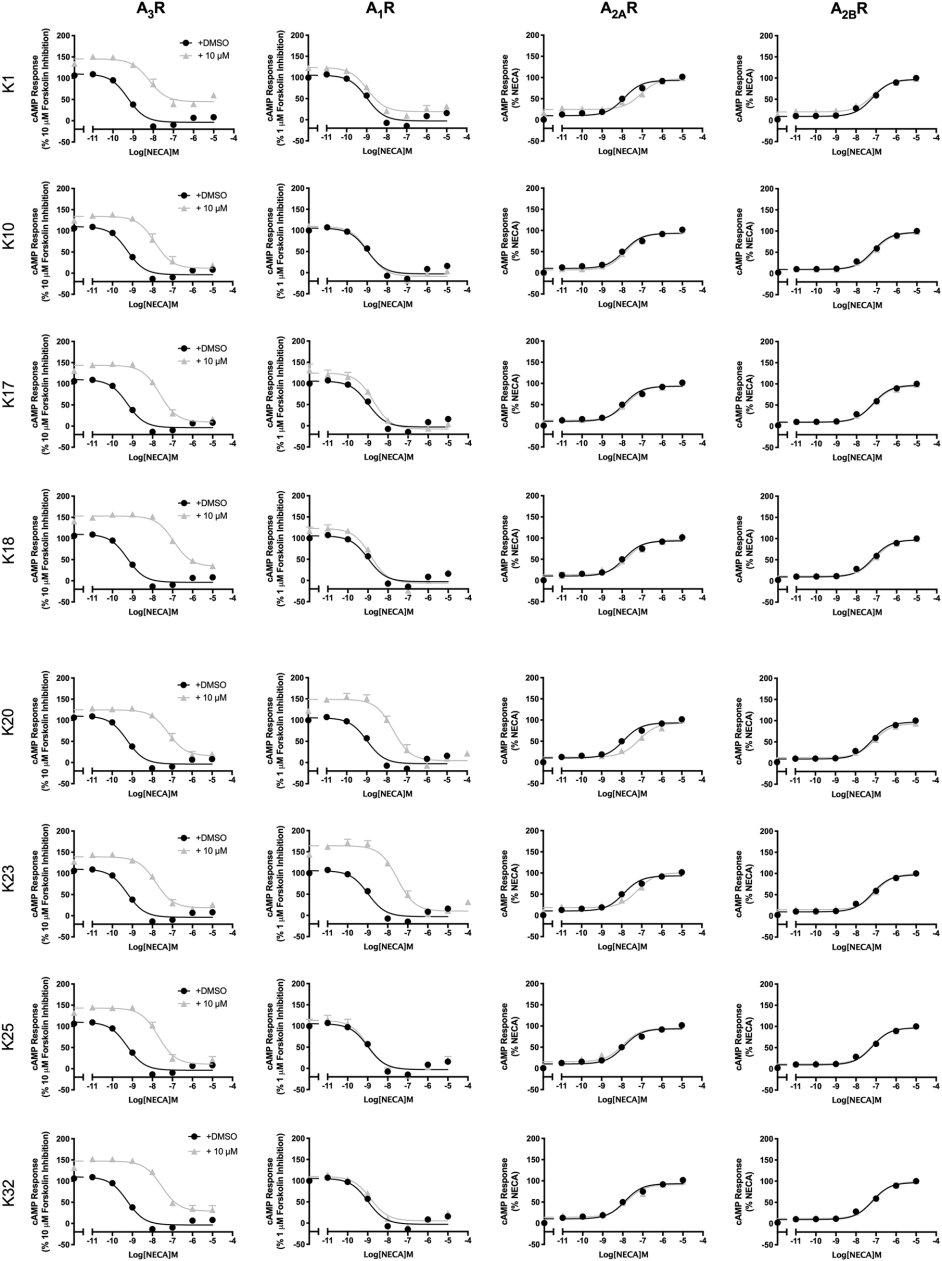
Characterisation of A_3_R antagonist at all AR subtypes. A_3_R Flp-In CHO cells or CHO-K1 cells (2000 cells/well) stably expressing one of the remaining AR subtypes were exposed to forskolin in the case of G_i_-coupled A_1_R and A_3_R (1 μM or 10 μM, respectively) or DMSO control in the case of G_s_-coupled A_2A_R and A_2B_R, NECA and test compound (10 μM) for 30 min and cAMP accumulation detected. All values are mean ± SEM expressed as percentage forskolin inhibition (A_1_R and A_3_R) or stimulation (A_2A_R and A_2B_R), relative to NECA. *n* ≥ 3 independent experimental repeats, conducted in duplicate.

The similarity of the different ARs has the consequence that many compounds display reduced selectivity. Using the A_3_R Flp-In CHO or CHO-K1 cells expressing A_1_R, A_2A_R or A_2B_R incubated with a single high concentration of antagonist (10 μM) and increasing concentrations of NECA identified K1, K10, K17, K18, K25 and K32 as A_3_R selective antagonists (Fig. 1). K20 and K23 were antagonists at both the A_1_R and A_3_R (Fig. 1 and Table 2). K1, K20 and K23 showed weak antagonism at the A_2A_R and none of the tested antagonist showed any antagonism of the NECA stimulated response at the A_2B_R. These selectivity findings agree with our previously published radioligand binding data (Lagarias et al*.*^19^) and are summarised in Table 2.

**Table 2:** Potency of NECA stimulated cAMP inhibition or accumulation as determined in Flp-In CHO or CHO-K1 cells expressing one of four ARs subtype (A_3_R, A_1_R, A_2A_R or A_2B_R).

	pIC_50_/pEC_50_^a^			
	A_3_R	A_1_R	A_2A_R	A_2B_R
NECA only	9.24 ± 0.1	8.98 ± 0.1	7.88 ± 0.1	7.24 ± 0.2
K1	8.01 ± 0.2****	8.97 ± 0.1	7.12 ± 0.1****	7.23 ± 0.2
K10	7.74 ± 0.2****	8.82 ± 0.1	7.84 ± 0.1	7.19 ± 0.2
K17	7.59 ± 0.1****	8.68 ± 0.1	7.76 ± 0.1	7.15 ± 0.2
K18	6.70 ± 0.1****	8.85 ± 0.1	7.75 ± 0.1	7.10 ± 0.2
K20	7.12 ± 0.2****	7.43 ± 0.1 ****	7.12 ± 0.1****	7.08 ± 0.1
K23	7.72 ± 0.1****	7.38 ± 0.1 ****	7.26 ± 0.1**	7.04 ± 0.2
K25	7.64 ± 0.1****	9.00 ± 0.1	7.98 ± 0.1	7.22 ± 0.2
K32	7.56 ± 0.1****	8.85 ± 0.1	7.80 ± 0.1	7.14 ± 0.2

Cells stably expressing A_3_R, A_1_R, A_2A_R or A_2B_R were stimulated with 10 μM forskolin (in the case of A_3_R and A_1_R), 10 μM tested compound/DMSO and increasing concentrations of NECA.

Data are expressed as mean ± SEM obtained in *n* = 5 independent experimental repeats, conducted in duplicate.

^a^Negative logarithm of NECA concentration required to produce a half-maximal response in the absence (NECA only) or presence of 10 μM compound at each AR subtype.

Statistical significance (*p < 0.05; **p < 0.01; ***p < 0.001; ****p < 0.0001) compared to ‘NECA only’ was determined by one-way ANOVA with Dunnett’s post-test.

### Characterisation of competitive antagonists at the A_3_R

**Figure 2:**
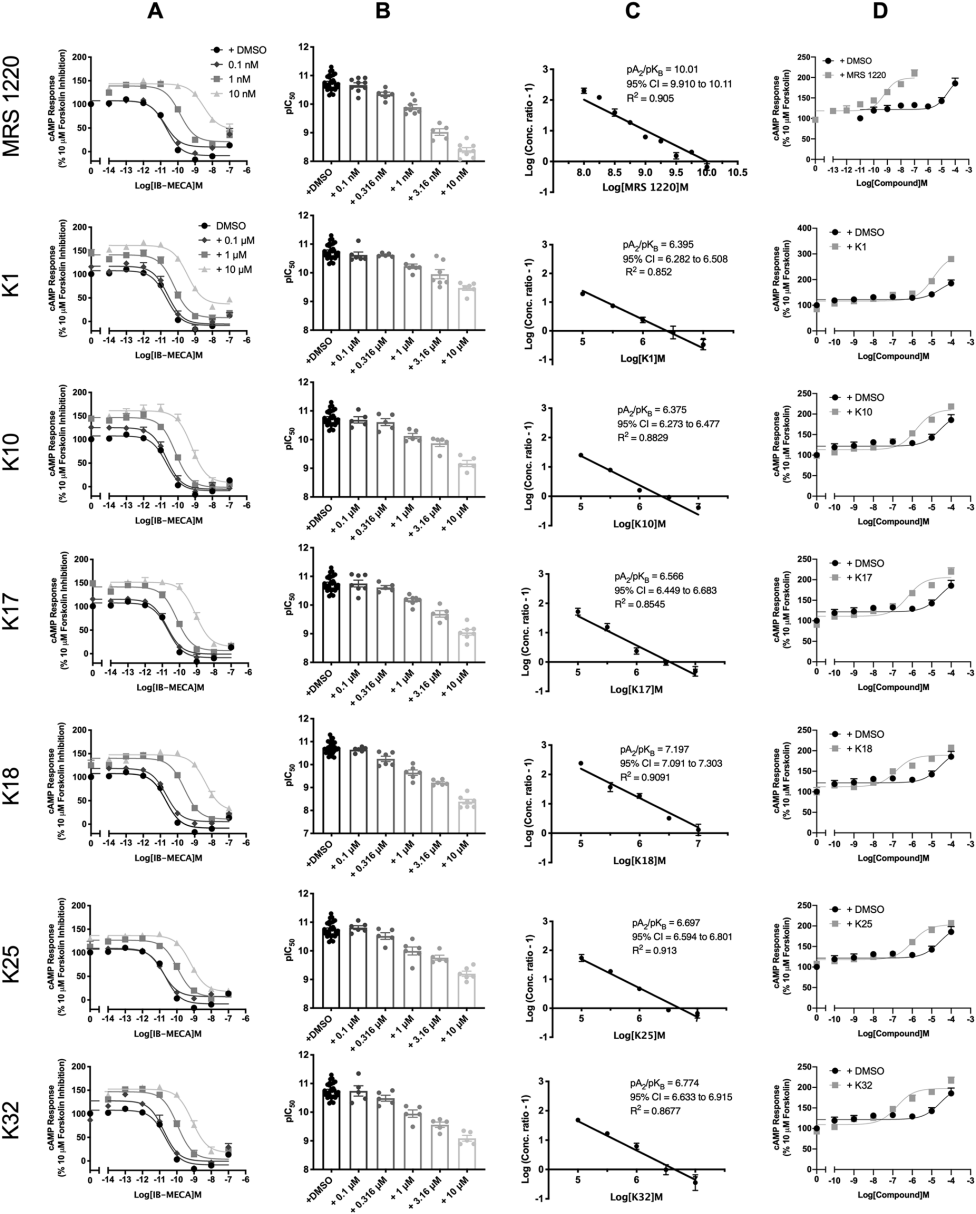
IB-MECA stimulated cAMP inhibition at WT A_3_R: activity of N-(9-chloro-2-furan-2-yl-[1,2,4]triazolo[1,5-c]quinazolin-5-yl)-2-phenylacetamide (MRS 1220) and potential antagonists. Flp-In-CHO cells (2000 cells/well) stably expressing WT A_3_R were exposed to forskolin 10 μM, IB-MECA and test compound/MRS 1220/DMSO control for 30 min and cAMP accumulation detected. (**A**) Representative dose response curves are shown as mean ± SEM expressed as percentage forskolin inhibition (10 μM) relative to IB-MECA. Key indicated in K1 is identical for all ‘K’ test compounds shown. (**B**) pIC_50_ values for individual repeats including half-log concentration are shown as mean ± SEM. (**C**) Schild analysis of data represented in (**A**) and (**B**). A slope of 1 indicates a competitive antagonist. The x-axis is expressed as − log (molar concentration of antagonist) giving a negative Schild slope. (**D**) Inverse agonism at the A_3_R. cAMP accumulation following a 30-min stimulation with forskolin (10 μM) and increasing concentrations of antagonist/DMSO control was determined in WT A_3_R expressing Flp-In-CHO cells. Representative dose response curves are shown as mean ± SEM expressed as percentage forskolin (10 μM), relative to IB-MECA.

All eight A_3_R antagonists were confirmed to antagonise (2S,3S,4R,5R)-3,4-dihydroxy-5-[6-[(3-iodophenyl)methylamino]purin-9-yl]-N-methyloxolane-2-carboxamide (IB-MECA) (Fig. 2 and Table 3) and preliminary data suggests this extends to NECA antagonism (Supplementary Fig. 4 and Supplementary Table 4) in a concentration-dependent manner. Schild analysis characterised K1, K10, K17, K18, K20, K23, K25 and K32 as competitive antagonists at the A_3_R (Schild slope not significantly different from unity, Fig. 2). K20 and K23 were also characterised as competitive antagonists at the A_1_R (Supplementary Fig. 5 and Supplementary Table 5).

**Table 3:** IB-MECA stimulated cAMP inhibition at WT A_3_R: activity of MRS 1220 and potential antagonists.

		WT A_3_R Flp-In-CHO						
		pIC_50_^a^	E_min_^b^	Basal ^c^	True Basal ^d^	Span ^e^	*n*	Inverse agonism pEC_50f._
IB-MECA only		10.72 ± 0.1	− 8.42 ± 2.6	107.7 ± 2.6	102.2 ± 2.9	116.1 ± 3.5	27	
MRS 1220	0.1 nM	10.67 ± 0.1	9.8 ± 3.5**	107.6 ± 3.7	99.7 ± 4.0	97.9 ± 4.9*	9	
	1 nM	9.90 ± 0.1****	20.9 ± 3.8***	139.0 ± 3.1****	124.8 ± 4.1**	118.1 ± 4.8	8	9.21 ± 0.2
	10 nM	8.39 ± 0.1****	46.7 ± 4.9****	143.6 ± 2.1****	133.8 ± 3.6****	96.9 ± 5.1*	8	
K1	0.1 μM	10.55 ± 0.1	− 5.4 ± 4.5	117.2 ± 4.3	105.9 ± 4.3	122.5 ± 5.9	6	
	1 μM	10.23 ± 0.1***	7.9 ± 4.5*	141.3 ± 3.7****	132.0 ± 6.6****	133.3 ± 5.6	7	4.93 ± 0.1
	10 μM	9.47 ± 0.1****	36.8 ± 4.4****	161.3 ± 2.9****	152.6 ± 5.4****	124.5 ± 5.1	6	
K10	0.1 μM	10.69 ± 0.1	− 5.2 ± 4.3	125.3 ± 4.0**	125.1 ± 6.3*	130.5 ± 5.6	5	
	1 μM	10.13 ± 0.1****	− 1.3 ± 4.4	146.7 ± 3.5****	140.4 ± 4.2****	148.1 ± 5.5**	5	5.81 ± 0.1
	10 μM	9.12 ± 0.1****	8.5 ± 6.4	161.1 ± 3.9****	150.0 ± 5.8****	152.6 ± 7.2****	5	
K17	0.1 μM	10.75 ± 0.1	− 0.9 ± 3.2	115.5 ± 3,3	111.8 ± 4.5	116.5 ± 4.5	7	
	1 μM	10.17 ± 0.1****	6.5 ± 3.8	141.7 ± 3.2****	131.7 ± 5.2****	135.3 ± 4.8*	7	6.24 ± 0.2
	10 μM	9.05 ± 0.1****	14.83 ± 5.2***	151.7 ± 3.0****	143.9 ± 6.1****	137.9 ± 5.8*	7	
K18	0.1 μM	10.65 ± 0.1	5.4 ± 2.6	118.6 ± 2.6	118.5 ± 4.1	113.1 ± 3.5	6	
	1 μM	9.65 ± 0.1****	10.7 ± 4.1*	140.1 ± 2.6****	125.6 ± 5.1**	129.4 ± 4.7	6	6.84 ± 0.2
	10 μM	8.38 ± 0.1****	28.0 ± 5.9****	147.7 ± 2.4****	138.6 ± 2.4****	119.7 ± 6.1	7	
K25	0.1 μM	10.81 ± 0.1	7.1 ± 2.5	109.5 ± 2.5	108.9 ± 3.3	102.4 ± 3.4	6	
	1 μM	10.12 ± 0.1****	6.7 ± 3.8	126.7 ± 2.9**	124.0 ± 4.1*	120.1 ± 4.6	5	6.01 ± 0.1
	10 μM	9.21 ± 0.1****	17.5 ± 3.2****	136.6 ± 1.8****	131.2 ± 4.1***	119.1 ± 3.5	6	
K32	0.1 μM	10.74 ± 0.1	− 0.6 ± 4.8	127.6 ± 4.9**	116.5 ± 6.0	128.2 ± 6.6	5	
	1 μM	9.95 ± 0.1****	3.6 ± 4.3	146.9 ± 3.4****	130.5 ± 5.8***	143.3 ± 5.3**	5	6.79 ± 0.2
	10 μM	9.09 ± 0.1****	17.7 ± 5.4***	152.3 ± 3.3****	140.2 ± 6.9****	134.6 ± 6.1	5	

Forskolin stimulated cAMP inhibition was measured in Flp-In-CHO stably expressing A_3_R following stimulation with 10 μM forskolin, compound at the indicated concentration and varying concentrations of IB-MECA.

^a^Negative logarithm of IB-MECA concentration required to produce a half-maximal response in the absence (IB-MECA only) or presence of 0.1, 1 or 10 μM compound.

^b^Minimum cAMP accumulation of IB-MECA as % of the 10 μM forskolin response relative to IB-MECA only; the lower plateau of the fitted sigmoidal dose response curve.

^c^The upper plateau of the fitted sigmoidal dose response curve corresponding to % of the 10 μM forskolin inhibition, relative to IB-MECA.

^d^The cAMP accumulation when stimulated with compound at the indicated concentration and 10 μM forskolin stimulation only.

^e^The difference between E_min_ and basal signaling.

^f^Value reported to determine inverse agonism: negative logarithm of compound concentration required to produce a half-maximal response.

Data are expressed as mean ± SEM obtained in *n* separate experiments. Inverse agonist experiments were conducted in 3 separate experiments. Statistical significance (*p < 0.05; **p < 0.01; ***p < 0.001; ****p < 0.0001) compared to ‘IB-MECA only’ was determined by one-way ANOVA with Dunnett’s post-test.

When comparing the activity of A_3_R selective antagonists (K10, K17, K18 and K25), K18 was the most potent, showed A_3_R specificity and higher A_3_R binding affinity (Table 2) and we propose it as our lead compound. The original competition binding experiments that identified the panel of antagonist was performed using [^3^H]2R,3R,4S,5R)-2-(2-hex-1-ynyl-6-methylaminopurin-9-yl)-5-(hydroxymethyl)oxolane-3,4-diol (HEMADO)^19^. To ensure the use of NECA or IB-MECA in our study did not influence characterisation of the compounds, we assessed the ability of K18 to antagonise cAMP inhibition by HEMADO at the A_3_R, and compared its potency to K17 (Supplementary Fig. 6 and Table 4). In this exploratory data, K18 again displayed higher potency than K17 at the A_3_R.

**Table 4:** Binding of compounds to the rat A_3_R.

	*pK*_*i*_	*n*
MRS 1220	6.74 ± 0.04	5
K1	6.07 ± 0.05	5
K10	4.19 ± 0.09	3
K17	4.60 ± 0.09	3
K18	4.60 ± 0.04	3
K20	5.71 ± 0.03	5
K23	5.93 ± 0.04	5
K25	6.37 ± 0.06	5
K32	4.05 ± 0.10	3

Equilibrium dissociation constant of MRS 1220 and K compounds as determined through NanoBRET ligand-binding (pK_i_).

**Figure 3:**
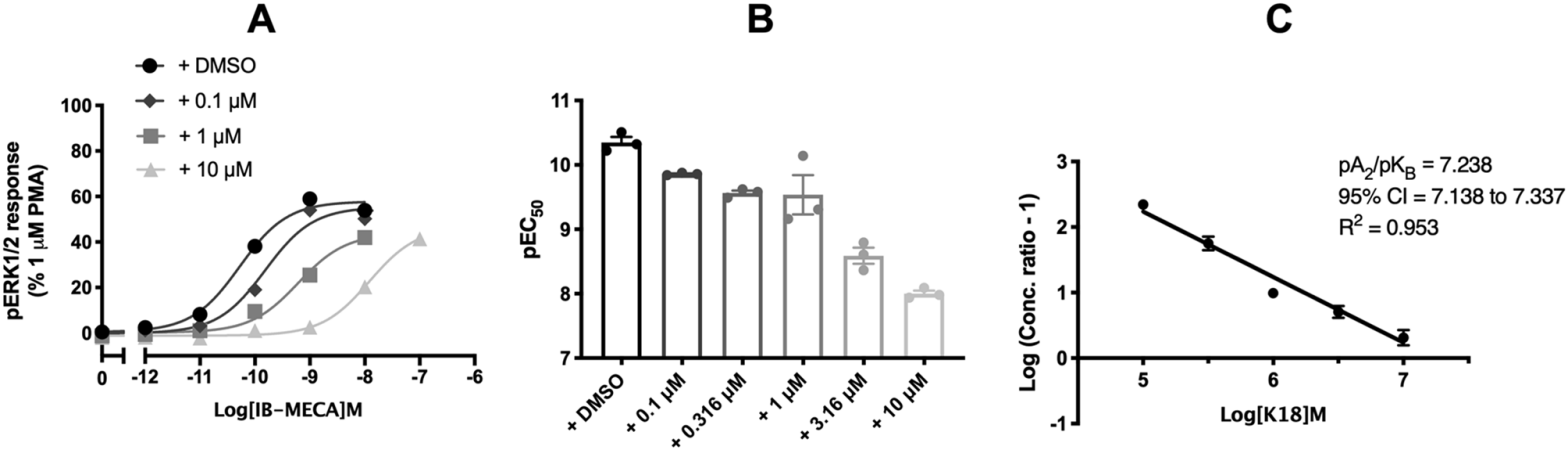
A_3_R constitutive activity and inverse agonism. K18 also reduced levels of agonist stimulated ERK1/2 phosphorylation. pERK1/2 was detected in Flp-In-CHO cells stably expressing A_3_R (2000 cells/well) stimulated for 5 min with (IB-MECA), with or without K18. (**A**) Representative dose response curves for IB-MECA with K18 at the indicated concentration or DMSO control shown as mean ± SEM expressed as % 1 μM PMA response. (**B**) pEC_50_ values for individual repeats are shown as mean ± SEM. (**C**) Schild analysis of data represented in (**A**) and (**B**).

In addition, we wanted to determine if K18 could also antagonise the activity of the A_3_R when an alternative downstream signalling component was measured; ERK1/2 phosphorylation (Fig. 3). In line with previously reported findings^11,20^, agonists at the A_3_R increased ERK1/2 phosphorylation after 5 min, with IB-MECA tenfold more potent than NECA (Supplementary Fig. 9) and preliminary data suggests this was entirely G_i/o_-mediated (pERK1/2 levels were abolished upon addition of PTX). K18 was able to antagonise A_3_R-mediated phosphorylation of ERK1/2 with the antagonist potency (pA_2_ values) not significantly different compared to the cAMP-inhibition assay (Fig. 3C).

### A_3_R constitutive activity and inverse agonism

We next determined if any of our competitive antagonist could function as inverse agonists of the A_3_R. In our hands, the A_3_R, when expressed in Flp-In-CHO cells, displays constitutive activity (Supplementary Fig. 7). All eight characterised A_3_R antagonists showed a concentration-dependent inverse agonism of the A_3_R when compared to DMSO control (Fig. 2) and were determined to be A_3_R dependent (Supplementary Fig. 8). This was also found to be the case for K20 and K23 at the A_1_R (Supplementary Fig. 5). Notably, DMSO showed a concentration-dependent elevation in cAMP accumulation above that of forskolin alone.

### Evaluation of the binding mode of K18 at A_3_R

**Figure 4:**
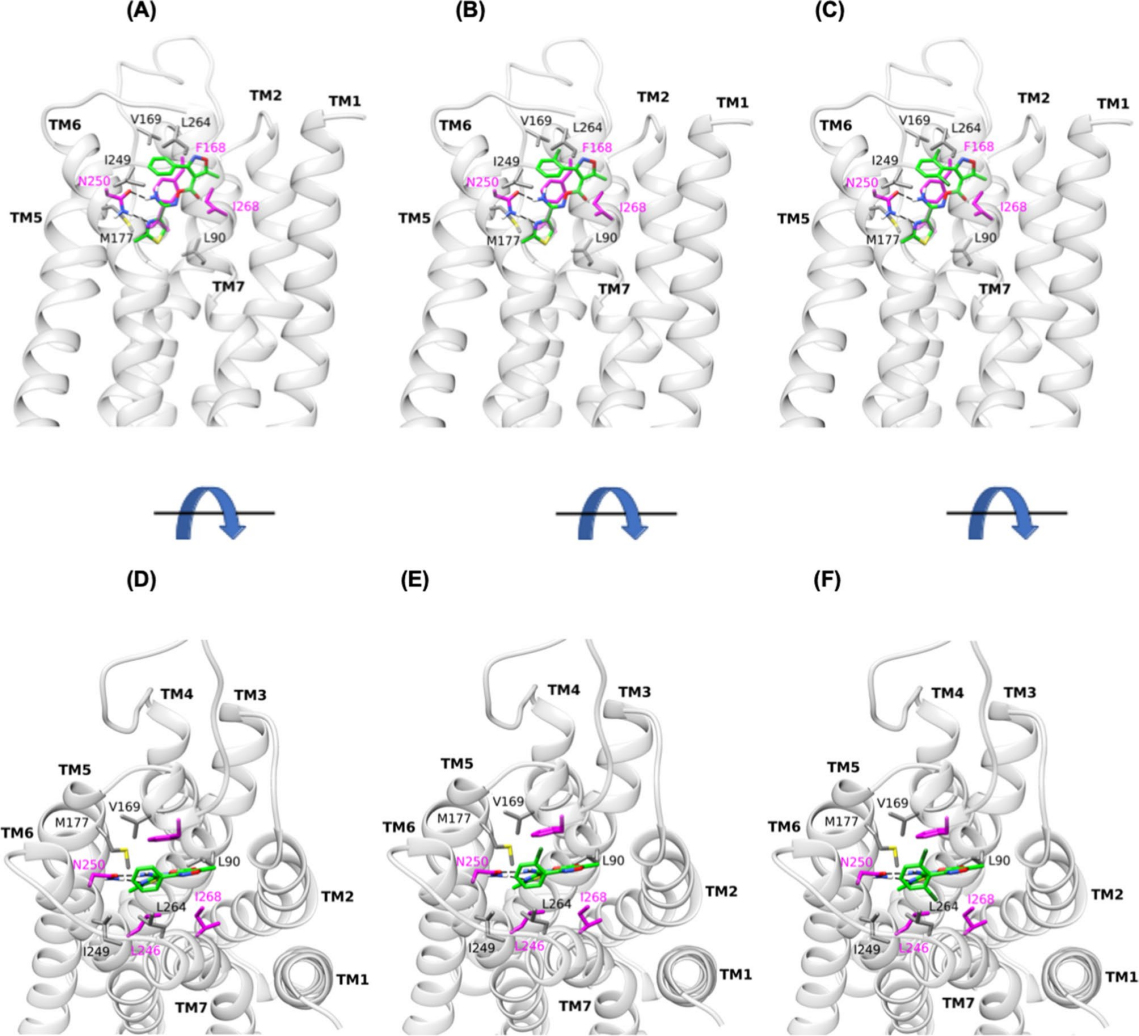
Orthosteric binding area average structure of WT A_3_R in complex with K5, K17 and K18 from MD simulations with Amber14ff. Side (**A**), top (**D**) view of K5 complex; side (**B**), top (**E**) view of K17 complex; side (**C**), top (**F**) view of K18 complex. Side chains of critical residues for binding indicated from the MD simulations are shown in sticks. Residues L90^3.32^, V169^5.30^, M177^5.40^, I249^6.54^ and L264^7.34^, in which carbon atoms are shown in grey, were confirmed experimentally; in residues F168^5.29^, L246^6.51^, I268^7.39^ and N250^6.55^ carbon atoms are shown in magenta; nitrogen, oxygen and sulfur atoms are shown in blue, red and yellow respectively.

We have previously investigated the binding mode of K18 at the human A_3_R using a homology model of the human A_3_R and simulations^21^. The computational model showed that the 3-(dichlorophenyl) group is positioned close to V169^5.30^, M177^5.40^, I249^6.54^ and L264^7.34^ of the A_3_R orthosteric binding site forming attractive vdW interactions. The amino group of the ligand is hydrogen-bonded to the amido group of N250^6.55^. The isoxazole ring is engaged in an aromatic *π-π* stacking interaction with the phenyl group of F168^5.29^ (Fig. 4). The thiazole ring is oriented deeper into the receptor favouring interactions with L246^6.51^, L90^3.32^ and I268^7.39^ (for full details see^19^).

**Figure 5:**
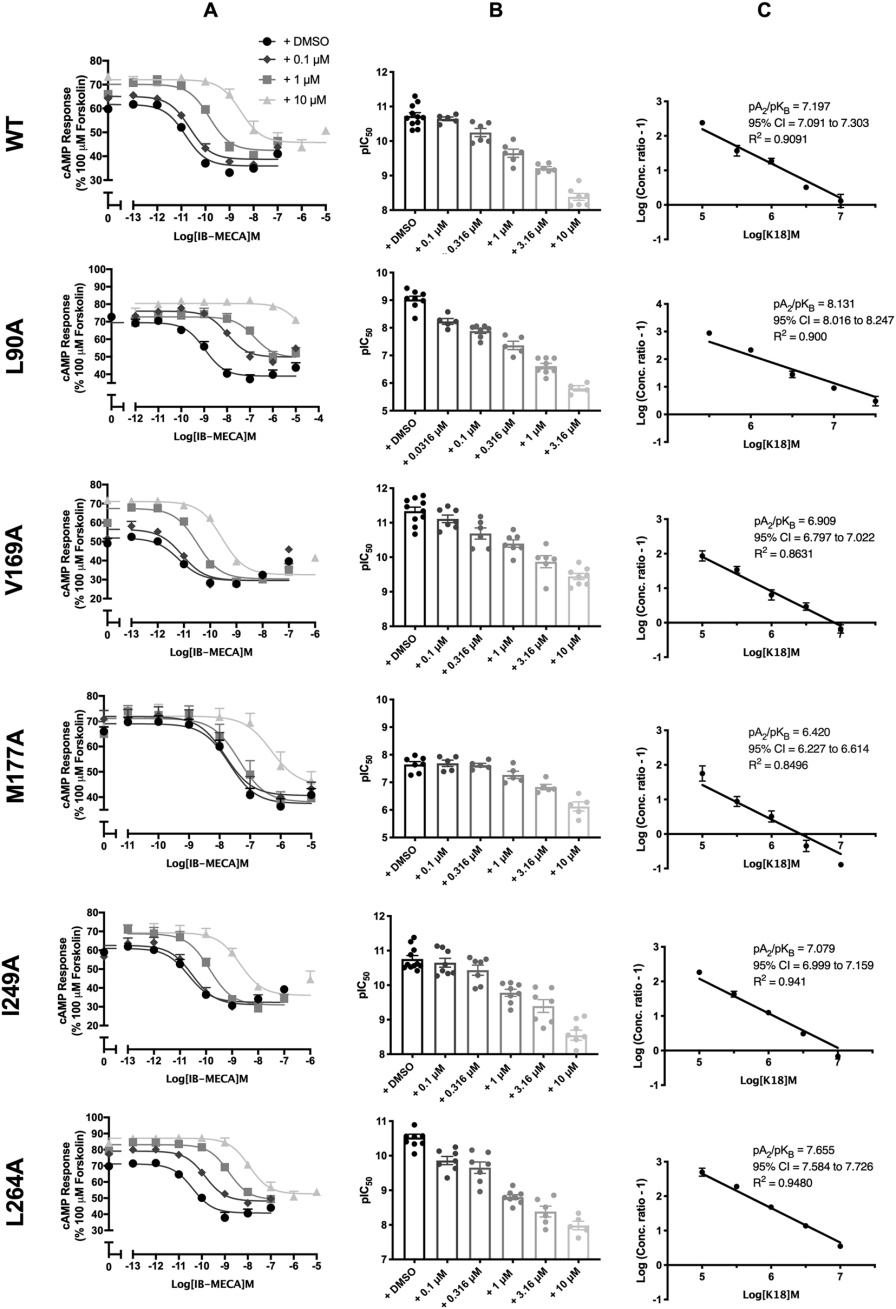
IB-MECA stimulated cAMP inhibition at WT or mutant A_3_R with increasing concentrations of K18. Flp-In-CHO cells (2000 cells/well) stably expressing WT or mutant A_3_R were exposed to forskolin 10 μM, IB-MECA and K18 at varying concentrations for 30 min and cAMP accumulation detected. (**A**) Representative dose response curves are shown as mean ± SEM expressed as percentage maximum forskolin response (100 μM). (**B**) pIC_50_ values for individual repeats including half-log concentration are shown as mean ± SEM. (**C**) Schild analysis of data represented in (**A**) and (**B**).

Of the identified residues predicted to mediate an interaction between K18 and the A_3_R, the ones which showed -according to the molecular dynamic (MD) simulations- the most frequent and the most important contacts were chosen for investigation and included amino acids L90^3.32^, F168^5.29^, V169^5.30^, M177^5.40^, L246^6.51^, I249^6.54^, N250^6.55^, L264^7.34^ and I268^7.39^ (Fig. 4). Site-directed mutagenesis was performed replacing each residue with an alanine in the A_3_R and expressed in the Flp-In-CHO™ cells lines. Each mutant was then screened for their ability to suppress forskolin-induced cAMP accumulation in response to NECA/IB MECA stimulation in the presence and absence of K18 and the detailed results are presented in Table 5 and Fig. 5. Mutation of residues F168^5.29^, L246^6.51^, N250^6.55^ and I268^7.39^ abolished agonist induced suppression of forskolin-induced cAMP accumulation and were discontinued in this study^22^. The affinity (pA_2_) of K18 at the WT and mutant A_3_R were compared in order to determine the potential antagonist binding site (Fig. 5, Table 5). As described previously^21^, K18 displayed no difference in affinity at I249A^6.54^ (compared to WT), whereas M177A^5.40^ and V169A^5.30^ were significantly smaller. Interestingly, we found an increase in the affinity for L90A^3.32^ and L264A^7.34^ when compared to WT. As would be expected, the K18 affinity at the A_3_R mutants was not different between agonists, confirming agonist independence (Supplementary Fig. 11). These experimental findings are reflected in our predicted binding pose of K18 at the WT A_3_R (Fig. 4)^21^.

**Table 5:** Antagonistic potency of K18 at A_3_R mutants.

	pIC_50_^a^	E_min_^b^	Basal^c^	True basal^d^	Span^e^	*n*
**+ DMSO**						
WT	10.73 ± 0.1	35.0 ± 1.6	60.1 ± 0.9	57.7 ± 1.3	25.1 ± 2.0	11
L90A	9.03 ± 0.1****	43.3 ± 3.2	73.2 ± 2.7***	71.5 ± 2.9***	29.9 ± 1.8	8
V169A	11.33 ± 0.1****	30.9 ± 1.7	55.3 ± 2.4	54.1 ± 2.5	24.3 ± 2.1	10
M177A	7.65 ± 0.1****	38.6 ± 2.7	70.2 ± 2.0*	66.7 ± 1.8	31.6 ± 2.0	7
I249A	10.76 ± 0.1	34.9 ± 2.2	62.6 ± 2.7	59.9 ± 2.6	27.7 ± 1.3	11
L264A	10.53 ± 0.1	41.1 ± 2.2	72.0 ± 2.3**	70.8 ± 2.6**	30.9 ± 2.2	9
**+ 0.1 μM K18**						
WT	10.64 ± 0.1	37.3 ± 1.8	63.0 ± 2.2	61.8 ± 2.6	25.8 ± 0.9	5
L90A	7.88 ± 0.1****	50.2 ± 3.4*	77.2 ± 2.6**	74.9 ± 2.8*	27.0 ± 3.1	7
V169A	11.11 ± 0.1 *	31.9 ± 1.8	62.6 ± 2.2	60.6 ± 3.1	30.6 ± 2.1	7
M177A	7.69 ± 0.1****	38.8 ± 2.5	70.9 ± 2.4	68.7 ± 2.3	32.1 ± 1.9	5
I249A	10.65 ± 0.1	35.6 ± 3.1	68.5 ± 3.3	67.0 ± 3.4	32.9 ± 1.3	8
L264A	9.86 ± 0.1***	45.7 ± 2.0	79.7 ± 2.7**	77.7 ± 3.0**	34.0 ± 2.8	7
**+ 1 μM K18**						
WT	9.65 ± 0.1	38.3 ± 2.4	67.4 ± 1.5	63.0 ± 1.8	29.1 ± 2.0	6
L90A	6.61 ± 0.1****	54.3 ± 3.6**	76.7 ± 3.2	73.5 ± 3.1	22.4 ± 2.6	8
V169A	10.40 ± 0.1****	31.9 ± 2.3	68.8 ± 1.7	66.5 ± 2.0	36.9 ± 2.5	7
M177A	7.27 ± 0.1****	40.0 ± 3.5	71.4 ± 2.4	66.3 ± 2.5	31.4 ± 2.0	5
I249A	9.78 ± 0.1	36.9 ± 3.2	76.3 ± 3.7	73.1 ± 3.8	39.3 ± 2.1*	8
L264A	8.80 ± 0.1****	47.9 ± 2.7	83.6 ± 2.1***	79.8 ± 2.4**	35.7 ± 3.0	8
**+ 10 μM K18**						
WT	8.38 ± 0.2	45.1 ± 1.7	72.0 ± 1.5	68.9 ± 1.6	26.9 ± 1.3	7
L90A	ND	59.9 ± 2.9**	81.7 ± 2.3	78.1 ± 2.4	22.8 ± 2.0	5
V169A	9.44 ± 0.1****	33.5 ± 1.8**	71.8 ± 1.6	69.2 ± 1.5	38.3 ± 2.0*	8
M177A	6.12 ± 0.2****	45.7 ± 3.2	72.1 ± 2.3	67.6 ± 2.4	26.6 ± 1.6	5
I249A	8.55 ± 0.2	36.6 ± 2.1*	78.0 ± 4.2	74.7 ± 4.2	38.7 ± 3.6*	8
L264A	7.98 ± 0.1	49.1 ± 3.1	85.4 ± 2.7*	82.8 ± 2.6**	33.7 ± 5.0	5

cAMP accumulation as measured in Flp-In-CHO cells stably expressing WT or mutant A_3_R following stimulation with 10 μM forskolin, varying concentrations of IB-MECA and ±K18 at the indicated concentration.

Data are expressed as mean ± SEM obtained in *n* separate experiments. All individual experiments were conducted in duplicate.

ND indicates an incomplete dose response curve due to the increased potency of K18 at this mutant.

^a^Negative logarithm of IB-MECA concentration required to produce a half-maximal response.

^b^Minimum cAMP accumulation of IB-MECA as %100 μM forskolin. The lower plateau of the fitted sigmoidal dose response curve.

^c^The upper plateau of the fitted sigmoidal dose response curve corresponding %100 μM forskolin.

^d^The cAMP accumulation when stimulated with 10 μM forskolin only + DMSO/K18 at the indicated concentration.

^e^The difference between E_min_ and basal signalling.

Statistical significance (*p < 0.05; **p < 0.01; ***p < 0.001; ****p < 0.0001) compared to WT IB-MECA stimulation ± K18 at each indicated concentration was determined by one-way ANOVA with Dunnett’s post-test.

We further investigated the suggested binding mode through comparatively modelled K5 and K17 binding at the A_3_R given their structural similarity with K18; K17 and K18 possess one and two chlorine atoms attached to the phenyl ring of the 3-phenyl-isoxazole moiety, respectively, whereas K5 has none (Fig. 4B,C). The suggested binding mode of K18 was further verified since the MM-PBSA calculated rankings in binding free energies were in agreement with experimental differences in binding affinities using radio-labelled assays and BRET (K5 < K17 < K18 < MRS 1220—Table 6) and antagonistic potencies with 5 having no chlorine atoms in 3-phenyl-isoxazolyl lacking any antagonistic potency. Finally, following MD simulations of compounds K26, K27, K29-K34 and K36-K39, we observed that K26 (and K34 for that matter) displayed a similar binding pose (Supplementary Fig. 10) to that of K18 (Fig. 4). K26, K34, K5, K17 and K18 have all similar binding affinities, measured by radio-labelled assays (Sup. Table 1). K26 and K34 contain the o-diphenyl-carbonyl instead of the 3-phenyl-isoxazole moiety in K5 but all three functionally showed weak antagonistic potency (> 1 μM, Supplementary Fig. 3), in contrast to K18 or K17 which contain a 3-(chlorophenyl)-isoxazolyl moiety or 3-(dichlorophenyl)-isoxazolyl moiety, respectively. These findings suggest that a more complex binding mode is present which will be investigated in the future, through synthetic installation of chlorine atoms in the ended phenyl ring of *o*-diphenyl-carbonyl moiety.

**Table 6:** Binding of K5, K17, K18 and MRS 1220 to the A_3_R orthosteric binding area.

	*E*_vdW_^a^	*E*_EL_^b^	Δ*G*_solv_^c^	Δ*G*_eff_^d^	*pK*_*B*_*/pK*_*i*_^e^		
					*S*child analysis^f^	NanoBRET^g^	Radioligand binding^h^
MRS 1220	**–** 64.6 ± 2.6	**–** 11.5 ± 2.5	39.2 ± 2.4	**–** 36.9 ± 3.6	10.07	9.99 ± 0.04	8.2–9.2
K5	**–** 42.0 ± 2.7	**–** 9.6 ± 5.2	30.8 ± 4.3	**–** 20.8 ± 4.3	ND	6.06 ± 0.09	5.02
K17	**–** 47.0 ± 2.4	**–** 8.8 ± 2.7	29.8 ± 2.9	**–** 25.9 ± 3.6	6.35	6.33 ± 0.03	5.38
K18	**–** 46.3 ± 2.9	**–** 7.5 ± 2.4	26.9 ± 3.1	**–** 26.9 ± 2.7	7.20	6.92 ± 0.10	6.05

Effective binding energies (Δ*G*_eff_) and energy components (*E*_vdW_, *E*_EL_, Δ*G*_solv_) in kcal mol^−1^ calculated using the MM-PBSA method.

^a^vdW energy of binding calculated using molecular mechanics.

^b^Electrostatic energy of binding calculated using molecular mechanics.

^c^Difference in solvation energy between the complex, the protein and the ligand, i.e. *G*_complex, solv_—(*G*_protein, solv_ + *G*_ligand, solv_).

^d^Effective binding free energy calculated as Δ*G*_eff_ = Δ*E*_ΜΜ_ + Δ*G*_sol_; in Table 6, Δ*E*_ΜΜ_ = *Ε*_vdW_^+^ *E*_EL_ (see “Materials and methods”).

^e^Equilibrium dissociation constant of MRS 1220, K5, K17 and K18 as determined through three independent experimental approaches: Schild analysis (pK_B_), NanoBRET (pK_i_) or radioligand binding (pK_i_).

^f^pK_B_ obtained through Schild analysis in A_3_R stably expressing Flp-In CHO cells.

^g^pK_i_ (mean ± SEM) obtained in NanoBRET binding assays using Nluc-A_3_R stably expressing HEK 293 cells and determined through fitting our “Kinetics of competitive binding, rapid competitor dissociation” model or in the case of MRS 1220 through fitting with the ‘Kinetics of competitive binding’ model with a determined K_on_ (*k*_3_) and K_off_ (*k*_4_) rate of 3.25 ± 0.28 × 10^8^ M^−1^ min^−1^ and 0.0248 ± 0.005 min^−1^, respectively.

^h^pK_i_ values previously published for K5, K17 and K18 (Lagarias et al*.,* 2018) or MRS 1220 (Stoddart et al*.,* 2015) through radioligand binding assays.

### Kinetics of A_3_R antagonists determined through BRET

**Figure 6:**
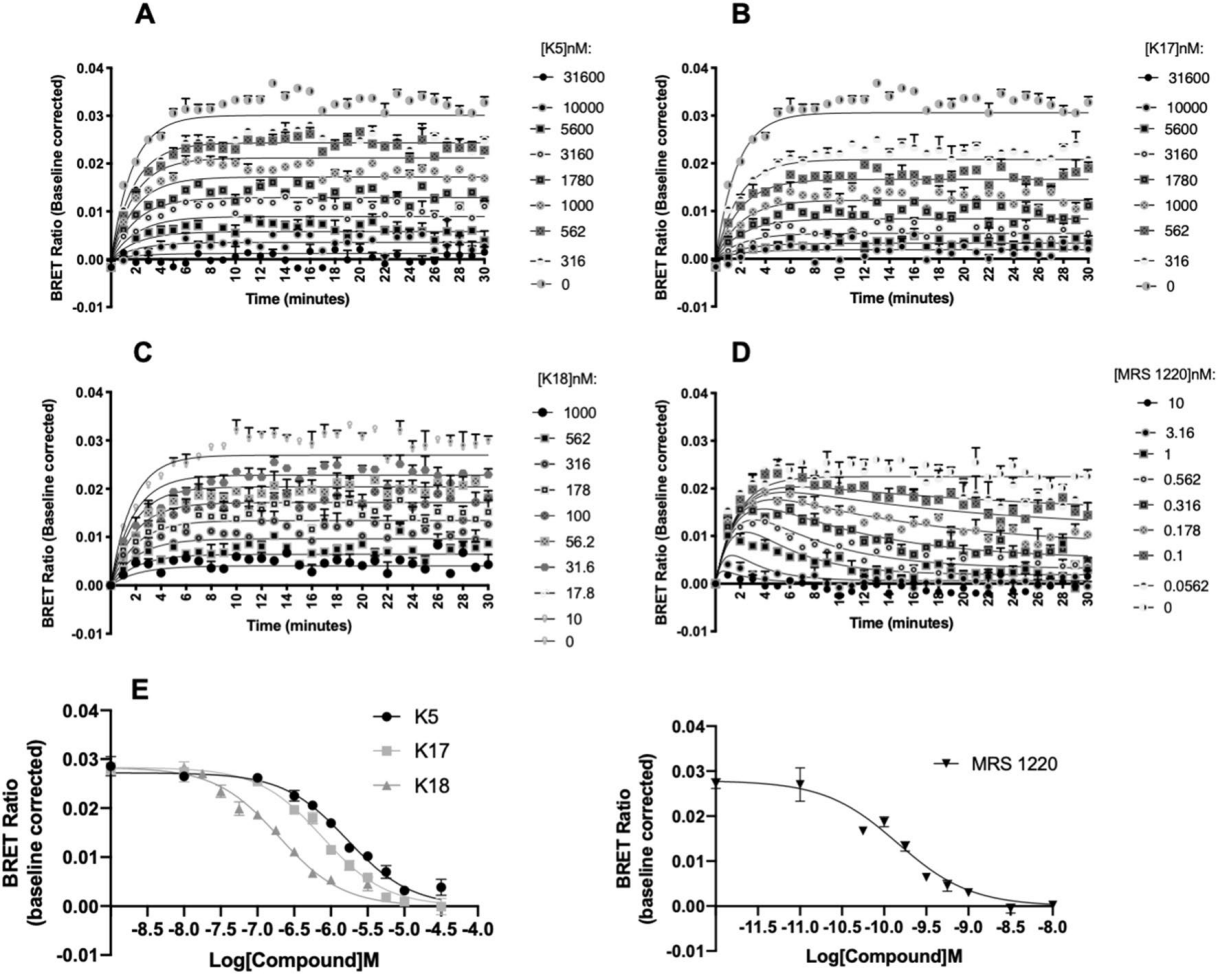
Inhibition of BRET between Nluc and CA200645 at the A_3_R by K5, K17, K18 and MRS 1220. HEK293 cells stably expressing Nluc-A_3_R were treated with 5 nM CA200645 and increasing concentrations of unlabelled compound (represented in nM) (**A**) K5, (**B**) K17, (**C**) K18 or (**D**) MRS 1220. For MRS 1220, this trace demonstrates a classic tracer ‘overshoot’, as has been previously described observed when the unlabelled compound has a slower off rate than the labelled CA200645 (K_off_ of 0.0248 ± 0.005 min^−1^ and 0.4397 ± 0.014 min^−1^ respectively) (Sykes et al*.*^24^, Motulsky and Mahan^28^). The data shown are representative of three independent experimental repeats (mean ± SEM) fitted with the appropriate model, as determined by statistical comparison between our new model (“Kinetics of competitive binding, rapid competitor dissociation”, derived in the Appendix I) (K5, K17 and K18) or the ‘kinetic of competitive binding’ model (built into Prism) for MRS 1220 (see “Materials and methods” for fitting procedure and statistical comparison method). (**E**) The resulting concentration dependent decrease in BRET ratio at 10 min was taken to calculate pK_i_ through fitting the Cheng-Prusoff equation^59^. Each data point represents mean ± SEM of five experiments performed in duplicate.

NanoBRET techniques have been successfully used to determine the real-time kinetics of ligand binding to GPCRs^23–25^. Here, we investigated the ability of the selective A_3_R antagonists MRS 1220, K17 or K18 to inhibit specific binding of the fluorescent A_3_R antagonist CA200645 to Nluc-A_3_R^26,27^. The kinetic parameters for CA200645 at Nluc-A_3_R were initially determined as K_on_ (*k*_1_) = 2.86 ± 0.89 × 10^7^ M^−1^ min^−1^, K_off_ (*k*_2_) = 0.4397 ± 0.014 min^−1^ with a K_D_ of 17.92 ± 4.45 nM. (Supplementary Fig. 12). Our MRS 1220 kinetic data was fitted with the original ‘kinetic of competitive binding’ model^28^ (built into GraphPad Prism 8.0) with a determined K_on_ (*k*_3_) and K_off_ (*k*_4_) rate of 3.25 ± 0.28 × 10^8^ M^−1^ min^−1^ and 0.0248 ± 0.005 min^−1^, respectively. This gave a residence time (RT) (RT = 1/K_off_) of 40.32 min. It was noticed in the analysis for K5, K17 and K18 that the fit in some cases was ambiguous and/or the fitted value of the compound dissociation rate constant was high (*k*_4_ > 1 min^−1^, corresponding to a dissociation *t*_1/2_ of < 42 s). In order to determine the reliability of the fitted *k*_4_ value, data were also analysed using an equation that assumes compound dissociation is too rapid for the dissociation rate constant to be determined reliably and the fits to the two equations were compared (“Kinetics of competitive binding, rapid competitor dissociation”, derived in the Appendix I). This model allowed estimate of the equilibrium binding affinity of the compound (*K*_i_) but not the binding kinetics of K5, K17 and K18 (Fig. 6 and Table 6). These affinity values were in agreement with those obtained via Schild analysis (except for K5) and were approximately tenfold higher than those determined through radioligand binding (Table 6). Notably, the order of affinity (K5 < K17 < K18) was consistent.

### Assessing the species selectivity of A_3_R antagonists

**Figure 7:**
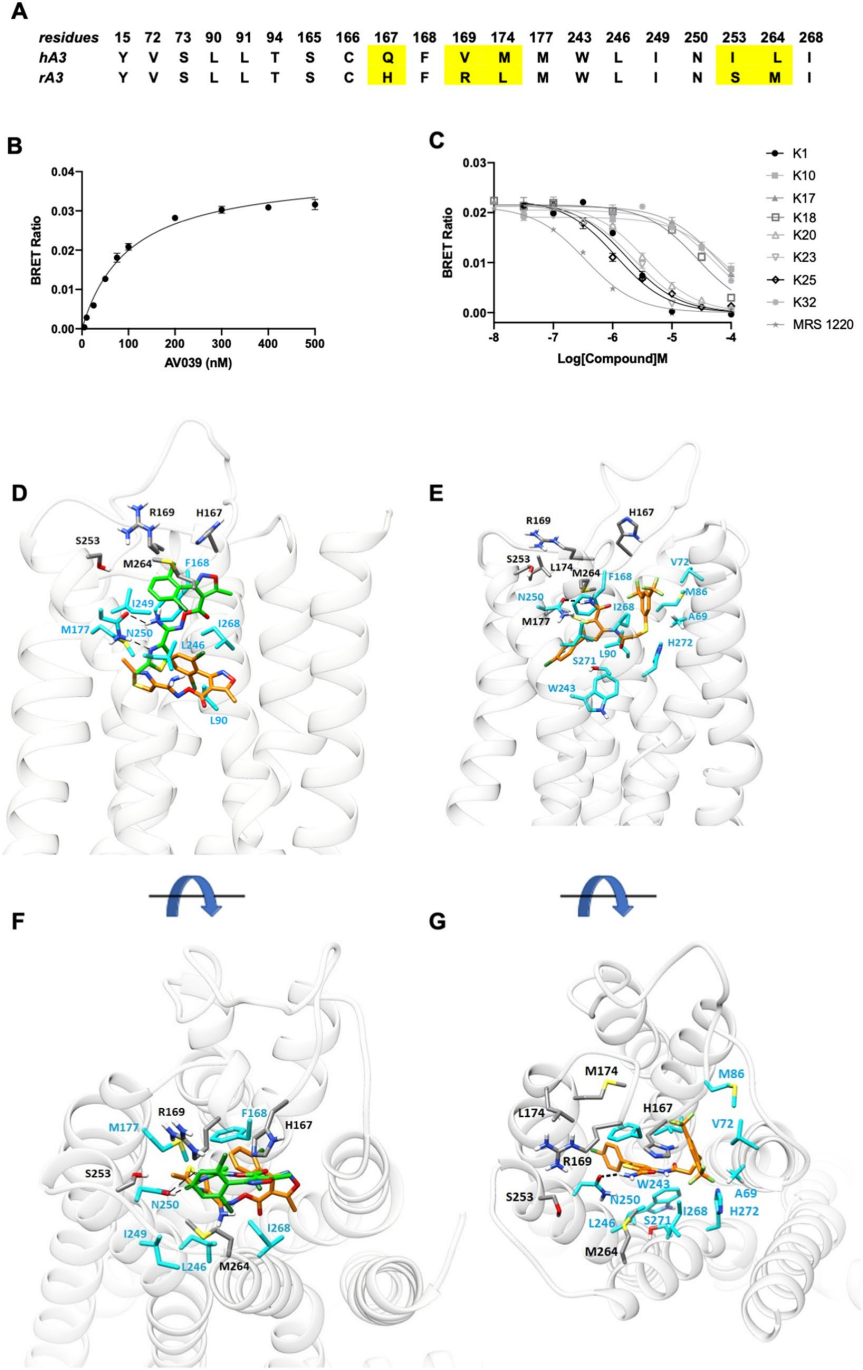
Pharmacological characterisation of K series of compounds at the rat A_3_R. (**A**) Comparison of the residues of the orthosteric binding area in human and rat A_3_Rs. (**B**) Saturation binding experiment with AV039 with a K_D_ of 102 ± 7.59 nM. (**C**) Inhibition of BRET between Nluc and AV039 at the rat A_3_R by MRS 1220 and K compounds. HEK293 cells stably expressing Nluc-rat A_3_R were treated with 100 nM AV039 and increasing concentrations of unlabelled compound. The resulting concentration dependent decrease in BRET ratio at 5 minutes was taken to calculate pK_i_ through fitting the Cheng-Prusoff equation^59^. Each data point represents mean ± SEM of *n* (*n* = 5 for MRS 1220, K1, K20, K23 and K25, *n* = 3 for K10, K17, K18 and K32) experiments, performed in duplicate. (**D**) Top and side (**E**) views of Rat A_3_R in complex with K18. Starting pose (carbons of the ligand in green), after 100 ns MD simulation (carbons of the ligand in orange). Light blue sticks show residues conserved with human A_3_R. M264^7.34^ most likely hampers K18 binding due to steric hindrance of the dichloro-phenyl group. (**F**) Top and side views (**G**) of the average structure of rat A_3_R in complex with K25 from 100 ns MD simulations (carbons of the ligand are shown in orange sticks and light blue sticks show residues in contact with K25). K25 was docked into the orthosteric site of the rat A_3_R using the GoldScore scoring function and the highest scoring pose was inserted in a hydrated POPE bilayer. The complexes were subjected to MD simulations with Amber14ff. and K25 adopts a potential binding pose within the orthosteric binding area.

There remains an issue as to understanding the precise role of the A_3_R. This is, in part due, to a lack of appropriate antagonists that are able to be tested in classical animal models such as rodent since only a few molecules (including the 6-phenyl-1,4-dihydropyridine MRS 1191 and the triazoloquinazoline MRS 1220) display cross species (human and rat) reactivity^29^. Comparison of residues of the binding area between human and rat A_3_R show that they differ in residues 167^5.28^, 169^5.30^, 176^5.37^, 253^6.58^, 264^7.34^ (Fig. 7). The scarcity of rat A_3_R antagonists prompted us to investigate if our characterised compounds were also potential antagonists at the rat A_3_R. Using a Nluc-tagged rat A_3_R expressing HEK 293 cell line, we conducted NanoBRET ligand-binding experiments whereby we determined the ability of our compounds to inhibit specific binding of the fluorescent antagonist AV039 to Nluc-rat A_3_R. As expected, AV039 was displaced by increasing concentrations of MRS 1220 (pK_i_ 6.788 ± 0.1) (Fig. 7 and Table 4). We found very weak binding of K17, K18, K10 and K32, with no binding detected below the concentration of 10 μM, whereas K1, K20, K23 and K25 were determined as potential rat A_3_R antagonists (pK_i_ 6.07 ± 0.04, 5.71 ± 0.03, 5.93 ± 0.04 and 6.37 ± 0.06, respectively) (Fig. 7 and Table 4). K25 had a higher binding affinity for the rat A_3_R when compared to the human A_3_R (Table 1) (pKi 6.37 ± 0.1 and 5.81, respectively).

MD simulations of the rat A_3_R (performed as described previously for the human A_3_R) suggested that the presence of M264^7.34^ most likely hampers K18 binding due to steric hindrance of the dichloro-phenyl group (Fig. 7). In contrast, MD simulations of K25 against rat A_3_R showed the formation of stable complex (Fig. 7). Here, the 2-amido group of the thiophene ring is hydrogen-bonded to the amido group of N250^6.55^. The thiophene ring forms aromatic π-π stacking interaction with F168^5.29^ and the 5-(p-chlorophenyl) is oriented deep in the binding area making contacts with L90^3.32^, L246^6.51^ and W243^6.48^. M264^7.34^ forces the large lipophilic moiety of the thiophene ring (3-NHCOCH_2_SPh(CF_3_)_2_) of K25 to locate close to TM2 favouring contacts with A69^2.61^, V72^2.64^, and I268^7.39^ (Fig. 7).

### Pharmacokinetic assessments of K18

The metabolic in vitro *t*_*1/2*_ (human liver microsomes, 0.1 mg/mL) of K18 (0.1 μM) was determined (0–60 min) as 24 min and the intrinsic clearance (CL_int_) calculated as 287.2 μl/min/mg (Supplementary Fig. 13). This was comparable to the reference compound verapamil and terfenadine (0.1 μM) with *t*_1/2_ determined as 35 and 12 min and CL_int_ as 200.1 or 581.1 μl/min/mg, respectively. Human plasma stability assessment determined the percentage of K18 (1 μM) remaining after 120 min as 90%, with a *t*_1/2_ of > 120 min. This is considerably higher than the reference compound propantheline (1 μM) which was determined to have a half-life of 55 min. The *t*_1/2_ of K18 (1 μM) in PBS (pH 7.4) over 240 min was determined as > 240 min, with 87% remaining at 240 min and was comparable to the reference compound propantheline (1 μM), with a determined *t*_1/2_ of > 240 min.

## Discussion

The search for an AR subtype specific compound often leads to compounds active at more than one of the AR subtypes because of the broad and similar orthosteric binding site of ARs^30^. Given that AR subtypes play distinct roles throughout the body, obtaining highly specific receptor antagonists and agonists is crucial. The virtual screening described by Lagarias et al*.*^19^ used a combination of a ligand-based and structure-based strategies based upon the experimental structure of A_2A_R in complex with the selective antagonist/inverse agonist ZM241385^31^ (PDB ID 3EML^32^), which has little affinity for A_3_R and 500- to 1000-fold selectivity for A_2A_R over A_1_R. Although, our high hit rate for A_3_R selective antagonist appears counter-intuitive, since the ligand-based virtual screening tool Rapid Overlay of Chemical Structures (ROCS) was used to predict structures similar to ZM241385,^19^.

Here, we present the pharmacological characterisation of eight A_3_R antagonists identified though virtual screening. Of these eight compounds, K10, K17, K18, K20, K23, K25 and K32 were determined to be competitive. Whereas K20 and K23 are antagonists at both the A_1_R and A_3_R, K10, K17, K18, K25 and K32 were are A_3_R selective antagonists. Indeed, we found no functional activity, or indeed binding affinity (< 30 μM), at the other AR subtypes. K1, K20 and K23 showed weak antagonism of the A_2A_R with no activity at the A_2B_R (Fig. 1, Table 2). These selectivity findings are in agreement with our radioligand binding data (Supplementary Table 1, and Lagarias et al*.*^19^ for K1–25, K28 and K35). However, a number of compounds previously determined to have micromolar binding affinity for A_3_R (K5, K9, K21, K22, K24, K26, K27 and K34), show no antagonistic potency in our initial functional screen. Further testing confirmed that these compounds are low potency antagonists and, although supporting the previously published radioligand binding data, confirmed the need for functional testing: not all compounds with binding affinity show high functional potency.

We show the A_3_R, when expressed in Flp-In-CHO cells, displays constitutive activity. Compounds which preferably bind to the inactive (R) state, decreasing the level of constitutive activity^33^ and in the case of a G_i/o_-coupled GPCR leading to an elevated cAMP, are referred to as inverse agonists. In this study all the A_3_R antagonists identified and both A_1_R antagonists (K20 and K23) were found to act as inverse agonists. We also report an elevation in cAMP accumulation when cells were stimulated with DMSO, which is concentration-dependent. Given that even low concentrations of DMSO have been reported to interfere with important cellular processes^34^, the interpretation of these data should be made with caution.

We show that the presence of a chloro substituent in the phenyl ring of 3-phenyl-isoxazole favoured A_3_R affinity, as following 0Cl < 1Cl < 2Cl i.e. K5 < K17 < K18. This theory is supported by both radioligand binding, NanoBRET ligand-binding and functional data. Moreover, MD simulations show that these compounds adopt a similar binding mode at the A_3_R orthosteric binding site, but the free-energy MM-PBSA calculations show that K18, having two chlorine atoms and an increased lipophilicity, leaves the solution state more efficiently and enters the highly lipophilic binding area. Importantly, substitution of the 1,3-thiazole ring in K17 with either a 2-pyridinyl ring (K32) or a 3-pyridinyl ring (K10) but not a 4-pyridinyl ring (K11) maintains A_3_R antagonistic potency. Also, substitution of 1,3-thiazole ring in K17 with the p-iodo-phenyl in K9 loses the antagonistic potency. Although we have not directly determined the effects of similar pyridinyl ring substitutions on the higher affinity antagonist K18, we suspect there would be no significant increase in the potency of K18 given the small changes we observed for K17.

For the first time, we demonstrated the utilisation of a new model which expands on the ‘Kinetic of competitive binding’ model^28^ (built into Prism 8.0) for fitting fast kinetics data obtained from NanoBRET experiments and assumes the unlabelled ligand rapidly equilibrates with the free receptor. Very rapid competitor dissociation can lead to failure of the fit, eliciting either an ambiguous fit (regression with Prism 8: “Ambiguous”, 2019) or unrealistically large K_3_ and K_4_ values. Whereas we were able to successfully fit the MRS 1220 kinetic data with the Motulsky and Mahan model due to its slow dissociation, fitting of K5, K17 and K18 kinetic data with this model often resulting in an ambiguous fit. Our new model, assuming fast compound dissociation, successfully fits the data and allows the determination of binding affinity. In the cases where the data were able to fit the Motulsky and Mahan model, the dissociation constant was higher (of the order of 1 min^−1^), indicating rapid dissociation. Although we found nearly a tenfold difference in determined binding affinity for MRS 1220, K5, K17 and K18 between BRET ligand binding and radioligand binding assays, we demonstrated the order of affinity remains consistent. Indeed, this is seen across all three experimental approaches: Schild analysis, NanoBRET ligand-binding assay and radioligand binding.

Combining MD simulations with mutagenesis data, we presented a final binding pose of K18 which appears to be within the orthosteric binding site, involving residues previously described to be involved in binding of A_3_R compounds^35^. We previously reported^22^ no detectable G_i/o_ response following co-stimulation with forskolin and NECA or IB-MECA for A_3_R mutants F168A^5.29^, L246A^6.51^, N250A^6.55^ and I268A^7.39^ and our findings are in line with previous mutagenesis studies investigating residues important for agonist and antagonist binding at the human A_3_R^36,37^. Through performing Schild analysis (results of which were used to inform modelling in Lagarias et al*.*^21^) we experimentally determined the effect of receptor mutation on antagonist affinity for L90A^3.32^, V169A/E^5.30^, M177A^5.40^, I249A^6.54^ and L264A^7.34^ A_3_R. The pA_2_ value for I249A^6.54^ A_3_R is similar to WT, whereas M177A^5.40^ and V169A^5.30^ are significantly smaller suggesting these residues appear to be involved in K18 binding. Interestingly we found an increase in K18 affinity at L90A^3.32^ and L264A^7.34^ when compared to WT. Our detailed MD simulations, results published elsewhere^21^ have investigated the selectivity profile of K18 and have demonstrated that K18 failed to bind A_1_R and A_2A_R due to a more polar area close to TM5, TM6 when compared to A_3_R.

We have also performed a preliminary pharmacokinetic assessment of K18 to assess its potential as a lead compound for future use in drug design. Based upon our initial findings K18 has a metabolic half-life and intrinsic clearance equivalent to verapamil and terfenadine. K18 is highly stable in human plasma. As such these studies suggest K18 is an ideal candidate for future investigations although a more detailed pharmacokinetic assessment is required.

Antagonists that are A_3_R-selective across species or at rat A_3_R alone are useful pharmacological tools to define the role of these receptors. The human and rat A_3_R display 72% homology^38^. The lack of rat A_3_R selective antagonists prompted us to investigate if our characterised A_3_R antagonists are potential antagonists at the rat A_3_R. We reported no binding of our lead A_3_R antagonist, K18, at the rat A_3_R and MD simulations suggest this is due to steric hinderance by M264^7.34^. This finding suggests that K18 may not only be A_3_R specific within the human ARs but may also be selective across species. Of the compounds that showed rat A_3_R binding (K1, K20, K23 and K25), K25 show the highest binding affinity and represents an interesting candidate for further investigation. MD simulations show K25 forms a stable complex with rat A_3_R and we suggest a potential binding pose.

In conclusion, we present findings of a unique scaffold (K18) which is both chemically and metabolically stable and as such can be used as a starting point for detailed structure–activity relationships and represents a useful tool that warrants further assessment. Furthermore, we introduce K25 as a potential rat A_3_R antagonist which also warrants further investigation.

## Materials and methods

### Cell culture and transfection

Cell lines were maintained using standard subculturing routines as guided by the European Collection of Cell Culture (ECACC) and checked annually for mycoplasma infection using an EZ-PCR mycoplasma test kit from Biological Industries (Kibbutz Beit-Haemek, Israel). All procedures were performed in a sterile tissue culture hood using aseptic technique and solutions used in the propagation of each cell line were sterile and pre-warmed to 37 °C. All cells were maintained at 37 °C with 5% CO_2_, in a humidified atmosphere. This study used CHO cell lines as a model due to the lack of endogenous AR subtype expression (Brown et al*.* 2008). CHO-K1-A_1_R, CHO-K1-A_2A_R, CHO-K1-A_2B_R and CHO-K1-A_3_R cells were routinely cultured in Hams F-12 nutrient mix (21,765,029, Thermo Fisher Scientific) supplemented with 10% Foetal bovine serum (FBS) (F9665, Sigma-Aldrich). Flp-In-CHO cells purchased from Thermo Fisher Scientific (R75807) were maintained in Hams F-12 nutrient mix supplemented with 10% FBS containing 100 μg/mL Zeocin Selection Antibiotic (Thermo Fisher Scientific).

Stable Flp-In-CHO cell lines were generated through co-transfection of the pcDNA5/FRT expression vector (Thermo Fisher Scientific) containing the gene of interest and the Flp recombinase expressing plasmid, pOG44 (Thermo Fisher Scientific). Transfection of cells seeded in a T25 flask at a confluency of ≥ 80% was performed using TransIT-CHO Transfection Kit (MIR 2174, Mirus Bio), in accordance with the manufacturer’s instructions. Here, a total of 6 μg of DNA (receptor to pOG44 ratio of 1:9) was transfected per flask at a DNA:Mirus reagent ratio of 1:3 (w/v). 48 h post-transfection, selection using 600 μg/mL hygromycin B (Thermo Fisher Scientific) (concentration determined through preforming a kill curve) was performed for two days prior to transferring the cells into a fresh T25 flask. Stable Flp-In-CHO cell lines expressing the receptor of interest were selected using 600 μg/mL hygromycin B whereby the media was changed every two days. Successful mutant cell line generation for non-signalling mutants were confirmed by Zeocin sensitivity (100 μg/mL).

The Nluc-tagged human A_3_R expressing HEK 293 cell line along with the Nluc-tagged rat A_3_R pcDNA3.1 + construct for the generation of stable Nluc-tagged rat A_3_R expressing HEK 293 cells were kindly gifted to us by Stephen Hill and Stephen Briddon (University of Nottingham). HEK 293 cells in a single well of 6-well plate (confluency ≥ 80%) were transfected with 2 μg of DNA using polyethyleneimine (PEI, 1 mg/ml, MW = 25,000 g/mol) (Polysciences Inc) at a DNA:PEI ratio of 1:6 (w/v). Briefly, DNA and PEI were added to separate sterile tubes containing 150 mM sodium chloride (NaCl) (total volume 50 μl), allowed to incubate at room temperature for 5 min, mixing together and incubating for a further 10 min prior to adding the combined mix dropwise to the cells. 48 h post-transfection, stable Nluc-rat A_3_R expressing HEK 293 cell were selected using 600 μg/mL Geneticin (Thermo Fisher Scientific) whereby the media was changed every two days. HEK 293 cell lines were routinely cultured in DMEM/F-12 GlutaMAX (Thermo Fisher Scientific) supplemented with 10% FBS (F9665, Sigma-Aldrich).

### Constructs

The human A_3_R originally in pcDNA3.1 + (ADRA3000000, cdna.org) was cloned into the pcDNA5/FRT expression vector and co-transfected with pOG44 to generate a stable Flp-In-CHO cell line. Mutations within the A_3_R were made using the QuikChange Lightening Site-Directed Mutagenesis Kit (Agilent Technologies) in accordance with the manufacturer’s instructions. The Nluc-tagged rat A_3_R pcDNA3.1 + construct, used in the generation of the stable Nluc-tagged rat A_3_R expressing HEK 293 cell line was kindly gifted to us by Stephen Hill and Stephen Briddon (University of Nottingham). All oligonucleotides used for mutagenesis were designed using the online Agilent Genomics ‘QuikChange Primer Design’ tool (detailed in Stamatis et al*.*^22^ (Table S4)) and purchased from Merck. All constructs were confirmed by in-house Sanger sequencing.

### Compounds

Adenosine, NECA, IB-MECA, HEMADO, DPCPX (8-cyclopentyl-1,3-dipropyl-7H-purine-2,6-dione) and MRS 1220 were purchased from Sigma-Aldrich and dissolved in dimethyl-sulphoxide (DMSO). CA200645, a high affinity AR xanthine amine congener (XAC) derivative containing a polyamide linker connected to the BY630 fluorophore, was purchased from HelloBio (Bristol, UK) and dissolved in DMSO. AV039, a highly potent and selective fluorescent antagonist of the human A_3_R based on the 1,2,4-Triazolo[4,3-a]quinoxalin-1-one linked to BY630^39^, was kindly gifted to us by Stephen Hill and Stephen Briddon (University of Nottingham). PMA was purchased from Sigma-Aldrich. Compounds under investigation were purchased from e-molecules and dissolved in DMSO. The concentration of DMSO was maintained to < 1.5% across all experiments (1.26% for all cAMP assays, 1% for pERK1/2 assays and 1.02% or 1.1% for NanoBRET ligand-binding experiments using CA200645 or AV039, respectively).

### cAMP accumulation assay

For cAMP accumulation (A_2A_R and A_2B_R) or inhibition (A_1_R or A_3_R) experiments, cells were harvested and re-suspended in stimulation buffer (PBS containing 0.1% BSA and 25 μM rolipram) and seeded at a density of 2000 cells per well of a white 384-well Optiplate and stimulated for 30 min with a range of agonist concentrations. In order to allow the A_1_R/A_3_R mediated G_i/o_ response to be determined, co-stimulation with forskolin, an activator of AC^40^, at the indicated concentration (depending on cell line) was performed. Testing of potential antagonists was performed in a competition experiment where cells received a co-stimulation with forskolin, agonist and compound/DMSO control, without test compound pre-incubation. cAMP levels were then determined using a LANCE cAMP kit as described previously^41^. In order to reduce evaporation of small volumes, the plate was sealed with a ThermalSeal film (Excel Scientific) at all stages.

### Phospho-ERK assay

ERK1/2 phosphorylation was measured using the homogeneous time resolved fluorescence (HTRF) Phospho-ERK (T202/Y204) Cellular Assay Kit (Cisbio Bioassays, Codolet, France) two-plate format in accordance with the manufacturer’s instructions. A_3_R expressing Flp-In-CHO were seeded at a density of 2000 cells per well of a white 384-well Optiplate and stimulated with agonist and test compounds for 5 min at 37 °C. Plate reading was conducted using a Mithras LB 940 (Berthold technology). All results were normalised to 5 min stimulation with 1 μM PMA, a direct protein kinase C (PKC). To determine if the measured pERK1/2 level was G_i_-mediated, we treated cells with Pertussis toxin (PTX) (Tocris Biosciences) for 16 h at 100 ng/mL prior to pERK assay.

### Radioligand binding

All pharmacological methods followed the procedures as described in the literature^42^. In brief, membranes for radioligand binding were prepared from CHO cells stably transfected with hAR subtypes in a two-step procedure. In the first step, cell fragments and nuclei were removed at 1000×*g* and then the crude membrane fraction was sedimented from the supernatant at 100,000×*g*. The membrane pellet was resuspended in the buffer used for the respective binding experiments and it was frozen in liquid nitrogen and stored at – 80 °C. For radioligand binding at the A_1_R, 1 nM [^3^H]CCPA was used, for A_2A_R 10 nM [^3^H]NECA and for A_3_R 1 nM [^3^H]HEMADO. Non-specific binding of [^3^H]CCPA was determined in the presence of 1 mM theophylline and in the case of [^3^H]NECA and [^3^H]HEMADO 100 μM R-PIA was used. *K*_i_ values from competition experiments were calculated using Prism (GraphPad Software, La Jolla, CA, USA) assuming competitive interaction with a single binding site. The curve fitting results (see Fig. 8 and 20) showed R^2^ values ≥ 0.99 for all compounds and receptors, indicating that the used one-site competition model assuming a Hill slope of n = 1 was appropriate.

### NanoBRET ligand-binding

Through the use of NanoBRET, real-time quantitative pharmacology of ligand-receptor interactions can be investigated in living cells. CA200645, acts as a fluorescent antagonist at both A_1_R and A_3_R with a slow off-rate^43^. Using an N-terminally NanoLuc (Nluc)-tagged A_3_R expressing cell line, competition binding assays were conducted. The kinetic data was fitted with the ‘kinetic of competitive binding’ model^28^ (Motulsky and Mahan, 1984; built into Prism) to determine affinity (pK_i_) values and the association rate constant (K_on_) and dissociation rates (K_off_) for unlabelled A_3_R antagonists. In several cases this model resulted in an ambiguous fit (Regression with Prism 8: “Ambiguous”, 2019). We developed a new model which expands on the ‘kinetic of competitive binding’ model to accommodate very rapid competitor dissociation, assuming the unlabelled ligand rapidly equilibrates with the free receptor. This method allows determination of compound affinity (pK_i_) from the kinetic data.

In order to identify if the characterised compounds also bound the rat A_3_R, we conducted competition binding assays using Nluc-tagged rat A_3_R expressing HEK 293 cells and the fluorescent compound AV039^39^ rather than xanthine based CA200645, which have previously been reported as inactive at rat A_3_R^44^. For both human and rat A_3_R experiments, filtered light emission at 450 nm and > 610 nm (640–685 nm band pass filter) was measured using a Mithras LB 940 and the raw BRET ratio calculated by dividing the 610 nm emission with the 450 nm emission. Here, Nluc on the N-terminus of A_3_R acted as the BRET donor (luciferase oxidizing its substrate) and CA200645/AV039 acted as the fluorescent acceptor. CA200645 was used at 25 nM, as previously reported^26^ and AV039 was used at 100 nM (pre-determined K_D_, 102 ± 7.59 nM). BRET was measured following the addition of the Nluc substrate, furimazine (0.1 μM). Nonspecific binding was determined using a high concentration of unlabelled antagonist, MRS 1220 at 10 nM or 10 μM, for human and rat A_3_R, respectively.

### Receptor binding kinetics data analysis

Specific binding of tracer vs time data was analysed using the Motulsky and Mahan method^28^ (built into Prism 8.0) to determine the test compound association rate constant and dissociation rate constant. Data were fit to the “Kinetics of competitive binding” equation in Prism 8.0 (GraphPad Software Inc, San Diego, CA):

$$\left[ {RL} \right]_{t} = \frac{{N\left[ L \right]k_{1} }}{{K_{F} - K_{S} }}\left[ {\frac{{k_{4} \left( {K_{F} - K_{S} } \right)}}{{K_{F} K_{S} }} - \frac{{k_{4} - K_{S} }}{{K_{S} }}e^{{ - K_{S} t}} + \frac{{k_{4} - K_{F} }}{{K_{F} }}e^{{ - K_{F} t}} } \right]$$

where,

$$K_{F} = 0.5\left\{ {K_{A} + K_{B} + \sqrt {\left( {K_{A} - K_{B} } \right)^{2} + 4\left[ L \right]\left[ I \right]k_{1} k_{3} } } \right\}$$

$$K_{S} = 0.5\left\{ {K_{A} + K_{B} - \sqrt {\left( {K_{A} - K_{B} } \right)^{2} + 4\left[ L \right]\left[ I \right]k_{1} k_{3} } } \right\}$$

$$K_{A} = \left[ L \right]k_{1} + k_{2}$$

$$K_{B} = \left[ I \right]k_{3} + k_{4}$$

[*RL*]_*t*_ is specific binding at time *t*, *N* the B_max_, [*L*] the tracer concentration, [*I*] the unlabelled competitor compound concentration, *k*_1_ the tracer association rate constant, *k*_2_ the tracer dissociation rate constant, *k*_3_ the compound association rate constant and *k*_4_ the compound dissociation rate constant.

Data were also analysed using an equation that assumes compound dissociation is too rapid for the dissociation rate constant to be determined reliably and the fits to the two equations compared (“Kinetics of competitive binding, rapid competitor dissociation”, derived in the Appendix I, Supplementary material). This equation assumes rapid equilibration between compound and receptor and consequently provides an estimate of the equilibrium binding affinity of the compound (*K*_i_) but not the binding kinetics of the compound. The equation is,

$$\left[ {RL} \right]_{t} = \frac{{N\left[ L \right]k_{1} \left( {1 - \rho_{I} } \right)}}{{k_{obs, + I} }}\left( {1 - e^{{ - k_{obs, + I} t}} } \right)$$

where *ρ*_*I*_ is fractional occupancy of receptors not bound by *L*:

$$\rho_{I} = \frac{\left[ I \right]}{{K_{I} + \left[ I \right]}}$$

and *k*_*obs*,+ *I*_ is the observed association rate of tracer in the presence of competitor, defined as,

$$k_{obs, + I} = \left[ L \right]k_{1} \left( {1 - \rho_{I} } \right) + k_{2}.$$

The fits to the two equations were compared statistically using a partial F-test in Prism 8.

### Pharmacokinetic assessments of K18

Preliminary pharmacokinetic assessments of K18 was out-sourced to Eurofins Panlabs (Missouri, U.S.A) and including tests for intrinsic clearance (human liver microsomes), plasma (human) stability and half-life in PBS. These tests were conducted in duplicate using a single concentration of K18 (0.1 μM or 1 μM) using the substrate depletion method. Here, the percentage of K18 remaining at various incubation times was detected using high-performance liquid chromatography mass spectrometry (HPLC–MS). Reference compounds (verapamil, terfenadine and propantheline) were supplied and tested alongside K18. The half-life (*t*_1/2_) was estimated from the slope (k) of percentage compound remaining (In(%K18 remaining)) versus time (*t*_*1/2*_ =—In(2)/k), assuming first order kinetics. The intrinsic clearance (CL_int,_ in μl/min/mg) was calculated according to the following formula:

$$CL_{int} = \frac{0.693}{{t_{1/2} *\left( {mg protein/\mu l} \right)}}$$

### Data and statistical analysis

All in vitro assay data was analysed using Prism 8.0 (GraphPad software, San Diego, CA), with all dose-inhibition or response curves being fitted using a 3-parameter logistic equation to calculate response range or E_max_ and IC/EC_50_. Experimental design ensured random distribution of treatments across 96/384-well plates to avoid systematic bias. Agonist stimulation alone was used as an intrinsic control across all experiments. Although initial screening of the 50 compounds was blinded, due to limitations in resources, it was not possible to perform all of our experiments in a blinded manner. Normalisation was used to control for unwanted sources of variation between experimental repeats.

In the rare cases where significant outliers were identified through the ROUT method (performed in Prism with Q set to 2% (defines the chance of falsely identifying one or more outliers)) (Statistics with Prism 7: “How to: Identify outliers”, 2019), these were excluded from data analysis and presentation. Dose-inhibition/dose–response curves were normalised to forskolin, expressed as percentage forskolin inhibition for G_i_-coupled A_1_R and A_3_R (1 μM or 10 μM, respectively) or stimulation for A_2A_R and A_2B_R (100 μM, representing the maximum cAMP accumulation of the system), relative to NECA/IB-MECA (agonist allowing comparison across AR subtypes and a selective A_3_R agonist, respectively). For cAMP experiments on A_3_R mutants, data was normalised to 100 μM forskolin, representing the maximum cAMP accumulation possible for each cell line. In the case of pERK1/2 response, normalisation was performed to PMA, a direct PKC activator providing the maximum pERK1/2 level of the system.

Schild analysis was performed to obtain pA_2_ values (the negative logarithm to base 10 of the molar concentration of an antagonist that makes it necessary to double the concentration of the agonist to elicit the original submaximal response obtained by agonist alone^45^) for the potential antagonists. In cases where the Schild slope did not differ significantly from unity, the slope was constrained to unity giving an estimate of antagonist affinity (p*K*_B_). pA_2_ and p*K*_B_ coincide when the slope is exactly unity. Through performing Schild analysis, whereby the pA_2_ is independent of agonist, we were able to experimentally determine the effect of receptor mutation on antagonist binding. The pA_2_ values obtained through conducting Schild analysis of K18 at WT and mutant A_3_R were compared in order to indicate important residues involved in K18 binding. Whereas an increase in the pA_2_ for a particular mutant when compared to WT suggested the antagonist was more potent, a decrease indicated a reduced potency.

All experiments were conducted in duplicate (technical replicates) to ensure the reliability of single values. The data and statistical analysis comply with the recommendations on experimental design and analysis in pharmacology^46^. Statistical analysis, performed using Prism 8.0, was undertaken for experiments where the group size was at least n = 5 and these independent values used to calculate statistical significance (*p < 0.05; **p < 0.01; ***p < 0.001; ****p < 0.0001) using a one-way ANOVA with a Dunnett’s post-test for multiple comparisons or Students’ t-test, as appropriate. Any experiments conducted n < 5 should be considered preliminary. Compounds taken forward for further investigation after our preliminary screening (n = 3) were selected based on a high mean cAMP accumulation (> 80%).

### Computational biochemistry

#### MD simulations

Preparation of the complexes between human A_3_R with K5, K17, K18 or MRS 1220 and rat A_3_R with K18 or K25 was based on a homology model of A_2A_R (see Appendix II in Supplementary material) and are detailed in Lagarias et al*.*^21^. Each ligand−protein complex was embedded in hydrated POPE bilayers. A simulation box of the protein–ligand complexes in POPE lipids, water and ions was built using the System Builder utility of Desmond (Desmond Molecular Dynamics System, version 3.0; D.E. Shaw Res. New York, 2011; Maest. Interoperability Tools, 3.1; Schrodinger Res. New York, 2012). A buffered orthorhombic system in 10 Å distance from the solute atoms with periodic boundary conditions was constructed for all the complexes. The MD simulations were performed with Amber14 and each complex-bilayer system was processed by the LEaP module in AmberTools14 under the AMBER14 software package^47^. Amber ff14SB force field parameters^48^ were applied to the protein, lipid14 to the lipids, GAFF to the ligands^49^ and TIP3P^50^ to the water molecules for the calculation of bonded, vdW parameters and electrostatic interactions. Atomic charges were computed according to the RESP procedure^51^ using Gaussian03^52^ and antechamber of AmberTools14^47^. The temperature of 310 K was used in MD simulations in order to ensure that the membrane state is above the main phase transition temperature of 298 K for POPE bilayers^53^. In the production phase, the relaxed systems were simulated in the NPT ensemble conditions for 100 ns. The visualization of produced trajectories and structures was performed using the programs Chimera^54^ and VMD^55^. All the MD simulations were run on GTX 1060 GPUs in lab workstations or on the ARIS Supercomputer.

#### MM-PBSA calculations

Relative binding free energies of the complexes between K5, K17, K18, MRS 1220 and A_3_R was estimated by the 1-trajectory MM-PBSA approach^56^. Effective binding energies (Δ*G*_eff_) were computed considering the gas phase energy and solvation free energy contributions to binding—see appendix II^21,22^.

#### Nomenclature of targets and ligands

Key protein targets and ligands in this article are hyperlinked to corresponding entries in https://www.guidetopharmacology.org, the common portal for data from the IUPHAR/BPS Guide to Pharmacology^57^, and are permanently archived in the Concise Guide to Pharmacology 2017/18^58^.

## Supplementary information

Supplementary file 1

## Data Availability

The data are available from the corresponding authors on reasonable request.

## Abbreviations

AR: Adenosine receptor
A_1_R: A^1^ adenosine receptor
A_2A_R: A_2A_ adenosine receptor
A_2B_R: A_2B_ adenosine receptor
A_3_R: A_3_ adenosine receptor
CA200645: Fluorescent xanthine amine congener
cAMP: Adenosine -3′,5′ cyclic monophosphate
CHO: Chinese hamster ovary
DMSO: Dimethyl sulfoxide
DPCPX: 8-Cyclopentyl-1,3-dipropyl-7H-purine-2,6-dione
ERK: Extracellular signal-regulated kinase
IB-MECA: (2S,3S,4R,5R)-3,4-dihydroxy-5-[6-[(3-iodophenyl)methylamino]purin-9-yl]-N-methyloxolane-2-carboxamide
HEMADO: (2R,3R,4S,5R)-2-(2-hex-1-ynyl-6-methylaminopurin-9-yl)-5-(hydroxymethyl)oxolane-3,4-diol
MRS 1220: N-(9-chloro-2-furan-2-yl-[1,2,4]triazolo[1,5-c]quinazolin-5-yl)-2-phenylacetamide
NECA: (2S,3S,4R,5R)-5-(6-aminopurin-9-yl)-N-ethyl-3,4-dihydroxyoxolane-2-carboxamide
Nluc: Nano-luciferase
Nluc-A_3_R: NanoLuc-labelled A_3_ adenosine receptor
PMA: Phorbol 12-myristate 13-acetate
MD: Molecular dynamic
MM-PBSA: Molecular Mechanics-Poisson Boltzmann Surface Area
SBDD: Structural-based drug design
vdW: Van der Waals
