# Supplementary material for: Pharmacological characterisation of novel adenosine A3 receptor antagonists
Source: Sci Rep. 2020 Nov 27;10:20781. doi: 10.1038/s41598-020-74521-y (PMC7695835; doi:10.1038/s41598-020-74521-y)
Supplement: Supplementary file 1 [file 41598_2020_74521_MOESM1_ESM.docx]

**SUPPORTING INFORMATION**

**Pharmacological Characterisation of Novel Adenosine A_3_ Receptor Antagonists**

Kerry Barkan, Panagiotis Lagarias, Margarita Stampelou, Dimitrios Stamatis, Sam Hoare, Dewi Safitri, Karl-Norbert Klotz, Eleni Vrontaki, Antonios Kolocouris and Graham Ladds

Correspondence and requests for materials should be addressed to: grl30@cam.ac.uk.

Supplementary Figures S1 – S13

Supplementary Tables S1 – S6

Appendix I and II

**SUPPORTING INFORMATION**

**Supplementary Figure 1.** **Screening for potential antagonists at the A_3_R.** cAMP accumulation was determined in Flp-In CHO cells stably expressing A_3_R (2000 cells/well) co-stimulated for 30 minutes with 10 μM forskolin, NECA at the pre-determined IC_80_ concentration (3.16 nM) and 1 μM of compound/DMSO control. An elevation in cAMP accumulation above that of 10 μM forskolin and NECA, as indicated by the grey dotted line, suggesting the compound is acting as an antagonist (black upwards arrow). Included is MRS 1220 (1 μM) as a positive control for competitive antagonist of A_3_R. A reduction of cAMP accumulation (black downwards arrow) could indicate a compound is acting as an agonist, causing cell toxicity or other non-specific activity. All values are mean ± SEM expressed as % 10 μM forskolin response (‘DMSO’) where *n* = 3 independent experimental repeats, conducted in duplicate. Grey downward arrow indicates potential antagonists with a cAMP level >80%.

| **Supplementary Table 1. Compounds with no apparent antagonist/agonist activity at the A_3_R.** Mean cAMP accumulation as measured in Flp-In CHO cells stably expressing A_3_R following stimulation with 10 μM forskolin only (DMSO) or NECA at the predetermined IC_80_ concentration and 1 μM test compound/DMSO control. Binding affinities were obtained through radioligand binding assays and chemical structures of new compounds tested against the A_1_R, A_2A_R and A_3_R are also included. | | | | | | | | |
| --- | --- | --- | --- | --- | --- | --- | --- | --- |
|  |  |  | **A_3_R Flp-In CHO** | | **Radioligand binding** | | |  |
|  |  |  |  | | **Ki (μM)^c^** | | |  |
|  | **Compound Name** | **Chemical structure** | **Mean ^a^** | **Mean**  **Change ^b^** | **A_3_R** | **A_1_R** | **A_2A_R** |  |
|  | NECA |  | 60.32 ±3.41 | - | - | - | - |  |
|  | **DMSO** |  | 100.00 ±1.15 | -35.73 | - | - | - |  |
| K2 | S05993^1^ | 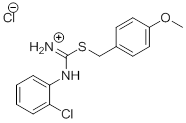 | 68.28 ±2.94 | -7.96 | 16.6 | >100 | 61.3 |  |
| K3 | SEW01061^1^ | 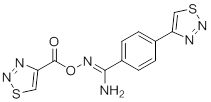 | 63.37 ±2.66 | -3.05 | >100 | >100 | >100 |  |
| K4 | SPB06895^1^ | 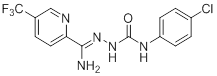 | 59.06 ±0.13 | 1.26 | >100 | >100 | >100 |  |
| K5 | SPB02733^1^ | 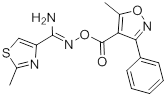 | 72.65 ±7.16 | -12.33 | **9.45** | >100 | 21.8 |  |
| K6 | KM08495^1^ | 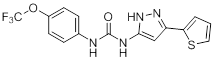 | 64.01 ±5.22 | -3.69 | 30.6 | >100 | >100 |  |
| K7 | HTS06244^1^ | 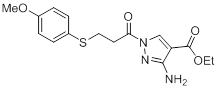 | 61.96 ±2.31 | -1.64 | 18.3 | >100 | >100 |  |
| K8 | KM03338^1^ |  | 51.56 ±5.80 | 8.757 | >100 | >100 | >100 |  |
| K9 | STK323059^1^ | 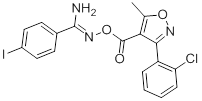 | 71.40 ±3.37 | 8.75 | **4.13** | **6.91** | >100 |  |
| K12 | STK441862^1^ | 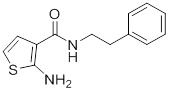 | 59.66 ±0.98 | 0.66 | 37.1 | >100 | >60 |  |
| K13 | STK448949^1^ | 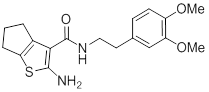 | 56.25 ±1.44 | 4.07 | 16.5 | >30 | >60 |  |
| K14 | STK450213^1^ | 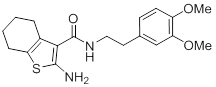 | 61.09 ±3.04 | -0.77 | 14.8 | >30 | >60 |  |
| K15 | STK106598^1^ | 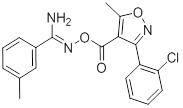 | 71.79 ±5.69 | -11.47 | 30.9 | >100 | >100 |  |
| K16 | Z56987720^1^ | 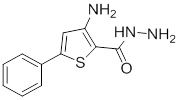 | 51.51 ±4.91 | 8.81 | 19.7 | >30 | 31.7 |  |
| K19 | RDR01677^1^ | 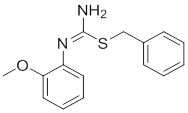 | 64.58 ±4.94 | -4.26 | >100 | >100 | >100 |  |
| K21 | HTS13009^1^ | 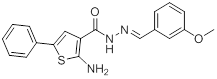 | 73.54 ±2.98 | -13.22 | **5.77** | >100 | **3.93** |  |
| K22 | HTS12882^1^ | 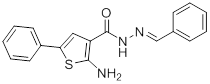 | 70.56 ±3.64 | -10.24 | **5.16** | 15.2 | **4.59** |  |
| K24 | GK01514^1^ | 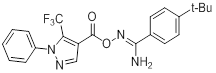 | 51.39 ±3.11 | 8.93 | **5.39** | **7.48** | >100 |  |
| K26 | 7709975 |  | 71.17 ±4.84 | -10.85 | **5.07** | >30 | 25.1 |  |
| K27 | 7709775 |  | 68.20 ±2.48 | -7.88 | 11.9 | >30 | 30.0 |  |
| K28 | GK00478^1^ | 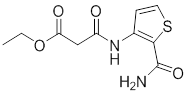 | 64.14 ±1.73 | -3.82 | >100 | 18.0 | 30.0 |  |
| K29 | 5687250 |  | 63.32 ±3.72 | -3.00 | >100 | >100 | >100 |  |
| K30 | 6169223 |  | 63.57±3.32 | -3.25 | >30 | >100 | >100 |  |
| K31 | 7721356 |  | 58.93 ±2.28 | 1.39 | 44.3 | >100 | >30 |  |
| K33 | STK300607 |  | 71.97 ±4.74 | -11.65 | >30 | >100 | >100 |  |
| K34 | 7713195 |  | 76.30 ±3.70 | -15.98 | **7.53** | >100 | >100 |  |
| K35 | Z1848163164^1^ | 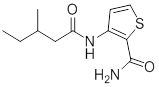 | 71.56 ±3.54 | -11.24 | 27.4 | **7.33** | >30 |  |
| K36 | STK710194 |  | 61.96 ±1.51 | -1.64 | >100 | >100 | >100 |  |
| K37 | 5685368 |  | 65.57 ±6.00 | -5.25 | >100 | >100 | >100 |  |
| K38 | 7968745 |  | 63.49 ±6.0 | -3.17 | >100 | >100 | >100 |  |
| K39 | 7712234 |  | 60.56 ±3.20 | -0.24 | 22.9 | >100 | >30 |  |
| ^1^Indicates previously published in Lagarias *et al.,* 2018 and is shown in grey  ^a^cAMP accumulation mean ± SEM expressed as %10 μM forskolin response where *n* ≥ 3 independent experimental repeats, conducted in duplicate. Potential antagonists were selected for further investigation based on a high mean cAMP accumulation (>80%).  ^b^Difference between the mean cAMP accumulation between ‘NECA’ and each compound expressed as %10 μM forskolin response  ^c^Binding affinity measured in three independent experiments. Bold denotes binding affinity < 10 μM. All compounds did not exhibit binding evidence to A_2B_R. | | | | | | | | |

**Supplementary Figure 2. Screening of potential antagonists at the A_3_R.** A_3_R Flp-In CHO cells (2000 cells/well) were exposed to forskolin 10 μM, NECA and DMSO or 1 μM test compound for 30 min and cAMP accumulation detected. All values are mean ± SEM expressed as percentage forskolin inhibition (10 μM) relative to NECA. *n* ≥ 3 independent experimental repeats, conducted in duplicate.

| **Supplementary Table 2. cAMP accumulation as measured in A_3_R Flp-In CHO cells following stimulation with 10 μM** **forskolin, varying concentrations of NECA and 1 μM test** **compound/DMSO control** | | | | | |
| --- | --- | --- | --- | --- | --- |
|  | **A_3_R Flp-In CHO** | | | |  |
|  | **pIC_50_ ^a^** | **E_min_ ^b^** | **Basal ^c^** | **True Basal ^d^** | **Span ^e^** |
| **NECA only** | 8.94 ±0.1 | -5.02 ±2.4 | 109.6 ±1.9 | 100.0 ±1.7 | 110.4 ±2.2 |
| **K1** | 8.46 ±0.1 | 10.5 ±4.5 | 126.9 ±3.3 | 115.7 ±2.8 | 118.0 ±3.1 |
| **K10** | 8.23 ±0.1 | 0.5 ±3.2 | 122.6 ±0.6 | 111.0 ±2.0 | 122.1 ±1.2 |
| **K11** | 8.88 ±0.1 | 4.7 ±4.3 | 126.5 ±3.2 | 109.1 ±2.0 | 121.9 ±5.2 |
| **K17** | 8.27 ±0.1 | 5.2 ±4.6 | 135.7 ±5.4 | 128.1 ±2.1 | 131.7 ±0.8 |
| **K18** | 7.79 ±0.1 | 9.5 ±4.8 | 139.2 ±3.7 | 127.2 ±3.2 | 130.9 ±7.1 |
| **K20** | 7.56 ±0.1 | 14.9 ±3.1 | 131.4 ±2.2 | 127.0 ±3.2 | 116.5 ±5.4 |
| **K23** | 7.85 ±0.1 | 20.4 ±3.2 | 130.5 ±2.6 | 122.7 ±3.9 | 110.7 ±2.7 |
| **K25** | 8.63 ±0.1 | -10.9 ±5.7 | 136.6 ±7.1 | 138.5 ±4.9 | 134.4 ±5.8 |
| **K32** | 8.31 ±0.1 | 10.5 ±7.1 | 129.4 ±4.5 | 113.1 ±5.6 | 118.9 ±7.1 |
| ^a^Negative logarithm of NECA concentration required to produce a half-maximal response in the absence (DMSO) or presence of 1 μM compound  ^b^Minimum cAMP accumulation of NECA as % 10 μM forskolin response, relative to NECA; the lower plateau of the fitted sigmoidal dose response curve  ^c^ The upper plateau of the fitted sigmoidal dose response curve corresponding to 100% of the 1 μM forskolin response  ^d^The cAMP accumulation when stimulated with 1 μM compound and 10 μM forskolin stimulation only  ^e^The difference between E_min_ and basal signalling | | | | | |

**Supplementary Figure 3. Determining functional activity of compounds with a micromolar binding affinity for A_3_R.** A_3_R Flp-In CHO cells (2000 cells/well) were exposed to forskolin 10 μM, NECA and DMSO or test compound at the indicated concentration for 30 min and cAMP accumulation determined. All values are mean ± SEM expressed as percentage forskolin inhibition (10 μM) relative to NECA. *n* = 3 independent experimental repeats, conducted in duplicate.

| **Supplementary Table 3. cAMP accumulation as measured in A_3_R Flp-In CHO cells following stimulation with 10 μM** **forskolin, varying concentrations of NECA and 1 μM or 10 μM test** **compound/DMSO control** | | | | | | |
| --- | --- | --- | --- | --- | --- | --- |
|  |  | **A_3_R Flp-In CHO** | | | |  |
|  |  | **pIC_50_ ^a^** | **E_min_ ^b^** | **Basal ^c^** | **True Basal ^d^** | **Span ^e^** |
| **NECA only** |  | 8.94 ±0.1 | -5.02 ±2.4 | 109.6 ±1.9 | 100.0 ±1.7 | 110.4 ±2.2 |
| **K5** | 1 μM | 9.06 ±0.2 | 2.3 ±8.2 | 115.3 ±7.0 | 102.5 ±10.1 | 113.1 ±10.5 |
|  | 10 μM | 7.93 ±0.1 | 39.3 ±5.6 | 148.3 ±3.1 | 142.1 ±9.3 | 108.9 ±6.2 |
| **K9** | 1 μM | 9.09 ±0.2 | 13.0 ±7.6 | 110.7 ±6.6 | 86.2 ±6.0 | 97.8 ±9.7 |
|  | 10 μM | 8.47 ±0.2 | 32.3 ±5.7 | 140.0 ±3.9 | 123.3 ±3.8 | 107.7 ±6.7 |
| **K11** | 1 μM | 8.88 ±0.1 | 4.7 ±4.3 | 126.5 ±3.2 | 109.1 ±2.0 | 121.9 ±5.2 |
|  | 10 μM | 7.83 ±0.2 | 39.4 ±7.5 | 139.3 ±5.0 | 119.8 ±9.2 | 99.9 ±8.2 |
| **K21** | 1 μM | 9.02 ±0.2 | 2.0 ±7.6 | 121.5 ±6.1 | 101.6 ±5.1 | 119.4 ±9.4 |
|  | 10 μM | 8.29 ±0.2 | 30.1 ±6.8 | 134.7 ±4.3 | 118.6 ±4.8 | 104.6 ±7.7 |
| **K22** | 1 μM | 9.07 ±0.2 | 0.4 ±7.1 | 121.3 ±5.8 | 101.9 ±8.4 | 120.9 ±8.8 |
|  | 10 μM | 8.12 ±0.2 | 41.6 ±7.1 | 139.1 ±4.2 | 124.5 ±8.2 | 97.5 ±8.0 |
| **K24** | 1 μM | 9.12 ±0.2 | 5.9 ±6.5 | 109.5 ±4.9 | 103.6 ±7.7 | 103.7 ±7.9 |
|  | 10 μM | 8.67 ±0.1 | 23.4 ±4.5 | 130.8 ±3.3 | 117.7 ±0.9 | 107.4 ±5.7 |
| **K26** | 1 μM | 9.10 ±0.2 | 5.7 ±6.2 | 118.2 ±4.8 | 98.9 ±8.8 | 112.5 ±7.6 |
|  | 10 μM | 8.49 ±0.1 | 41.5 ±5.7 | 158.8 ±3.9 | 143.0 ±5.9 | 117.3 ±6.6 |
| **K27** | 1 μM | 9.29 ±0.2 | 21.5 ±7.4 | 112.0 ±6.8 | 93.1 ±6.7 | 90.5 ±9.7 |
|  | 10 μM | 8.51 ±0.2 | 44.9 ±5.3 | 146.3 ±3.7 | 133.2 ±3.0 | 101.4 ±6.2 |
| **K34** | 1 μM | 9.04 ±0.2 | 21.1 ±8.7 | 116.2 ±6.9 | 97.1 ±2.1 | 95.1 ±10.8 |
|  | 10 μM | 8.49 ±0.2 | 46.2 ±6.7 | 154.9 ±4.7 | 143.6 ±7.6 | 108.7 ±7.8 |
|  | | | | | | |

^a^Negative logarithm of NECA concentration required to produce a half-maximal response in the absence (DMSO) or presence of 1 μM or 10 μM compound

^b^Minimum cAMP accumulation of NECA as % 10 μM forskolin response, relative to NECA; the lower plateau of the fitted sigmoidal dose response curve

^c^The upper plateau of the fitted sigmoidal dose response curve corresponding to 100% of the 10 μM forskolin response

^d^The cAMP accumulation when stimulated with 1 μM or 10 μM compound and 10 μM forskolin stimulation only

^e^The difference between E_min_ and basal signalling

**C**

**B**

**A**

**Supplementary Figure 4. NECA stimulated cAMP inhibition at WT A_3_R: activity of potential antagonists.** Flp-In-CHO cells (2000 cells/well) stably expressing WT A_3_R were exposed to forskolin 10 μM, NECA and test compound/DMSO control for 30 min and cAMP accumulation detected. **A**) Representative dose response curves are shown as mean ± SEM expressed as percentage forskolin inhibition (10 μM) relative to NECA. **B**) pIC_50_ values are shown as mean ± SEM. **C**) Schild analysis of data represented in **A/B**. A slope of 1 indicates a competitive antagonist**.** The x-axis is expressed as -log (molar concentration of antagonist) giving a negative Schild plot slope.

| **Supplementary Table 4. cAMP accumulation as measured in Flp-In-CHO stably expressing A_3_R following stimulation with 10 μM** **forskolin, compound at the indicated concentration and varying concentrations of NECA** | | | | | | |
| --- | --- | --- | --- | --- | --- | --- |
|  |  | **WT A_3_R Flp-In-CHO** | | | |  |
|  |  | **pIC_50_ ^a^** | **E_min_ ^b^** | **Basal ^c^** | **True Basal ^d^** | **Span ^e^** |
| **NECA only** | | 8.94 ±0.1 | -5.02 ±2.4 | 109.6 ±1.9 | 100.0 ±1.7 | 110.4 ±2.2 |
| **K1** | 0.1 μM | 8.73 ±0.2 | -8.38 ±5.4 | 118.3 ±3.3 | 107.2 ±3.0 | 117.0 ±2.5 |
|  | 1 μM | 8.46 ±0.1 | 10.5 ±4.5 | 126.9 ±3.3 | 115.7 ±2.8 | 118.0 ±3.1 |
|  | 10 μM | 7.80 ±0.1 | 54.1 ±4.5 | 148.5 ±6.6 | 145.7 ±2.4 | 96.9 ±3.3* |
| **K10** | 0.1 μM | 8.76 ±0.2 | -6.0 ±5.6 | 112.9 ±1.9 | 98.2 ±3.1 | 109.7 ±2.8 |
|  | 1 μM | 8.23 ±0.1 | 0.54 ±3.2 | 122.6 ±0.6 | 111.0 ±2.0 | 122.1 ±1.2 |
|  | 10 μM | 7.15 ±0.1 | 19.2 ±4.5 | 131.7 ±1.8 | 121.9 ±2.6 | 112.6 ±2.6 |
| **K17** | 0.1 μM | 9.00 ±0.1 | -5.5 ±4.8 | 122.6 ±1.9 | 115.0 ±4.4 | 124.6 ±2.5 |
|  | 1 μM | 8.27 ±0.1 | 5.2 ±4.6 | 135.7 ±5.4 | 128.1 ±2.1 | 131.7 ±0.8 |
|  | 10 μM | 7.43 ±0.1 | 18.7 ±5.2 | 146.6 ±3.3 | 138.6 ±3.1 | 131.2 ±4.0 |
| **K18** | 0.1 μM | 8.58 ±0.1 | 6.1 ±5.5 | 131.1 ±5.4 | 122.3 ±2.1 | 127.5 ±4.7 |
|  | 1 μM | 7.79 ±0.1 | 9.5 ±4.8 | 139.2 ±3.7 | 127.2 ±3.2 | 130.9 ±7.1 |
|  | 10 μM | 6.61 ±0.1 | 28.3 ±5.5 | 148.5 ±2.6 | 143.1 ±1.3 | 121.7 ±1.4 |
| **K20** | 0.1 μM | 8.38 ±0.1 | 2.2 ±4.1 | 119.6 ±3.9 | 122.3 ±2.1 | 117.9 ±3.4 |
|  | 1 μM | 7.56 ±0.1 | 14.9 ±3.1 | 131.4 ±2.2 | 127.0 ±3.2 | 116.5 ±5.4 |
|  | 10 μM | 6.68 ±0.1 | 23.6 ±3.8 | 130.2 ±1.9 | 143.1 ±1.3 | 106.8 ±5.5 |
| **K23** | 0.1 μM | 8.36 ±0.1 | 7.5 ±3.4 | 119.5 ±3.2 | 117.3 ±3.2 | 112.2 ±1.3 |
|  | 1 μM | 7.85 ±0.1 | 20.4 ±3.2 | 130.5 ±2.6 | 122.7 ±3.9 | 110.7 ±2.7 |
|  | 10 μM | 7.35 ±0.1 | 25.9 ±3.5 | 135.3 ±2.3 | 129.3 ±4.9 | 109.2 ±7.3 |
| **K25** | 0.1 μM | 9.10 ±0.2 | -2.7 ±6.2 | 122.0 ±7.0 | 126.7 ±6.5 | 126.6 ±4.4 |
|  | 1 μM | 8.63 ±0.1 | -10.9 ±5.7 | 136.6 ±7.1 | 138.5 ±4.9 | 134.4 ±5.8 |
|  | 10 μM | 7.54 ±0.2 | 17.9 ±7.0 | 151.7 ±3.5 | 148.5 ±5.4 | 136.5 ±2.5 |
| **K32** | 0.1 μM | 9.22 ±0.1 | -1.3 ±4.9 | 124.5 ±5.4 | 107.4 ±5.4 | 114.9 ±8.4 |
|  | 1 μM | 8.62 ±0.1 | 30.5 ±4.9 | 144.8 ±4.1 | 132.3 ±4.3 | 115.4 ±6.8 |
|  | 10 μM | 7.53 ±0.1 | 42.6 ±3.0 | 144.7 ±2.9 | 126.5 ±3.0 | 91.7 ±6.8 |
| ^a^Negative logarithm of NECA concentration required to produce a half-maximal response in the absence (NECA only) or presence of 0.1, 1 or 10 μM compound  ^b^Minimum cAMP accumulation of NECA as % 10 μM forskolin response relative to NECA response; The lower plateau of the fitted sigmoidal dose response curve  ^c^The upper plateau of the fitted sigmoidal dose response curve corresponding to % 10 μM forskolin inhibition, relative to NECA  ^d^The cAMP accumulation when stimulated with compound at the indicated concentration and 10 μM forskolin stimulation only  ^e^The difference between E_min_ and basal signaling | | | | | | |


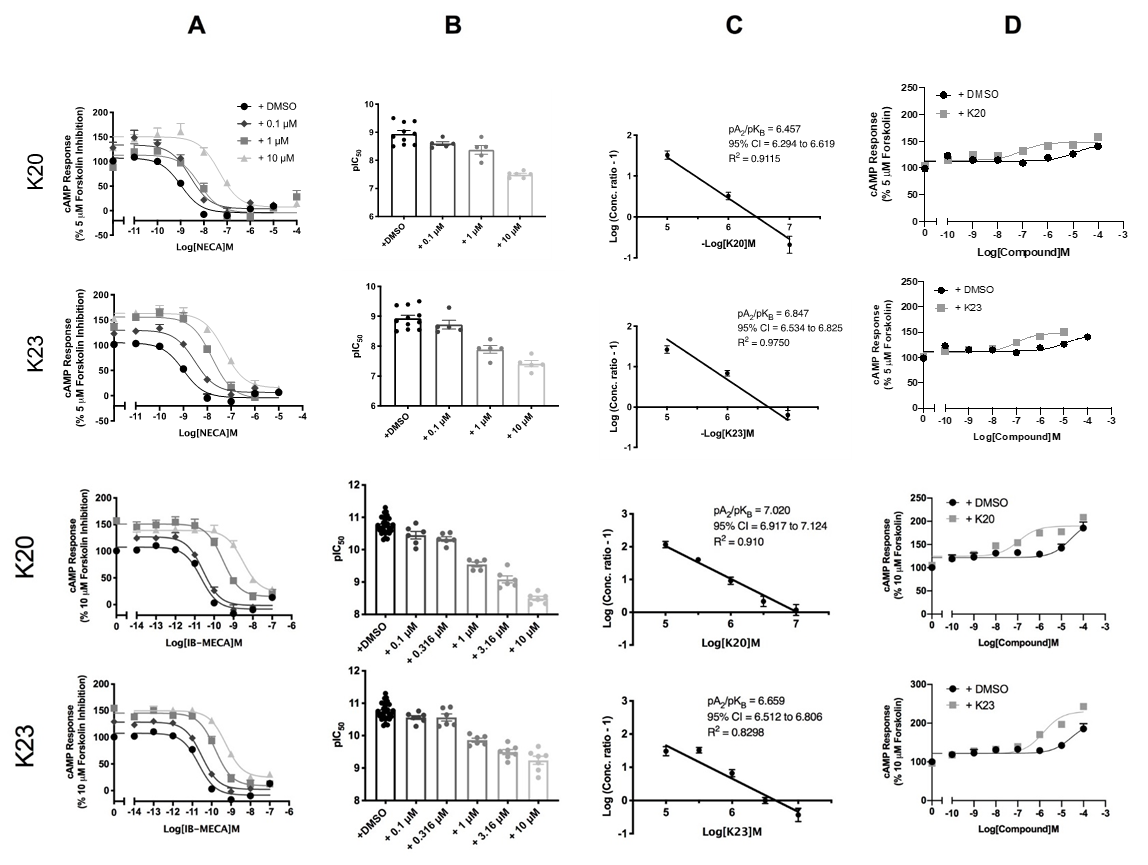


**Supplementary Figure 5. Agonist stimulated cAMP inhibition at A_1_R or A_3_R: activity of K20 and K23.** CHO-K1 cells (2000 cells/well) stably expressing A_1_R or Flp-In-CHO cells stably expressing A_3_R were exposed to forskolin (5 or 10 μM, respectively), agonist and test compound/DMSO control for 30 min and cAMP accumulation detected. **A**) Representative dose response curves are shown as mean ± SEM expressed as percentage forskolin inhibition relative to NECA/IB-MECA. **B**) pIC_50_ values for individual repeats including half-log concentration are shown as mean ± SEM. **C**) Schild analysis of data represented in **A/B**. A slope of 1 indicates a competitive antagonist. The x-axis is expressed as -log (molar concentration of antagonist) giving a negative Schild plot slope. . **D**) K20 and K23 show inverse agonism at A_1_R and A_3_R. cAMP accumulation following a 30-minute stimulation with forskolin (5 or 10 μM, respectively) and increasing concentrations of antagonist/DMSO control was determined. Representative dose response curves are shown as mean ± SEM expressed as percentage forskolin (10 μM), relative to IB-MECA.

| **Supplementary Table 5. cAMP accumulation as measured in CHO-K1 stably expressing A_1_R of**  **or Flp-In-CHO cells stably expressing A_3_R following stimulation with forskolin (5 or 10 μM, respectively), compound at the indicated concentration and varying concentrations of agonist NECA/IB-MECA** | | | | | | | |
| --- | --- | --- | --- | --- | --- | --- | --- |
| **A_1_R CHO-K1** | | | | | | | **Inverse agonism** |
|  |  | **pIC_50_ ^a^** | **E_min_ ^b^** | **Basal ^c^** | **True Basal ^d^** | **Span ^e^** | **pEC_50_^f^** |
| **NECA only** |  | 8.94 ±0.1 | -5.1 ±1.0 | 107.5 ±3.4 | 100.0 ±1.16 | 111.4 ±4.0 |  |
| **K20** | 0.1 μM | 8.68 ±0.1 * | -5.3 ±9.3 | 130.1 ±2.6 | 110.9 ±4.4 | 135.4 ±6.7 |  |
|  | 1 μM | 8.37 ±0.1 **** | -3.7 ±3.8 | 106.6 ±8.5 | 113.6 ±18.1 | 98.6 ±5.6 | 7.08 ±0.2 |
|  | 10 μM | 7.38 ±0.1 **** | 8.1 ±2.0** | 136.6 ±2.3** | 140.7 ±6.4* | 153.5 ±12.1*** |  |
| **K23** | 0.1 μM | 8.52 ±0.1 **** | 3.3 ±5.6 | 131.6 ±5.1 | 117.6 ±3.2 | 128.3 ±7.2 |  |
|  | 1 μM | 8.00 ±0.1 **** | 0.1 ±6.3 | 132.8 ±3.8* | 119.8 ±3.2* | 149.5 ±15.5* | 6.99 ±0.2 |
|  | 10 μM | 7.49 ±0.1 **** | 12.2 ±4.7* | 178.3 ±16.2**** | 170.2 ±13.6**** | 166.0 ±15.0** |  |
| **WT A_3_R Flp-In-CHO** | | | | | | |  |
| **IB-MECA only** |  | 10.72 ±0.1 | -8.42 ±2.6 | 107.7 ±2.6 | 102.2 ±2.9 | 116.1 ±3.5 |  |
|  | 0.1 μM | 10.45 ±0.1 | -1.6 ±4.1 | 127.1 ±4.2**** | 124.5 ±6.4** | 128.7 ±5.6 |  |
| **K20** | 1 μM | 9.54 ±0.1**** | 15.1 ±6.5** | 151.0 ±4.3**** | 145.3 ±9.8**** | 135.9 ±7.4 | 6.96 ±0.2 |
|  | 10 μM | 8.50 ±0.1**** | 23.3 ±7.8**** | 139.0 ±3.4**** | 128.0 ±8.0** | 115.7 ±8.2 |  |
|  | 0.1 μM | 10.56 ±0.1 | -2.4 ±3.3 | 128.6 ±3.1**** | 123.4 ±5.2** | 126.2 ±4.4 |  |
| **K23** | 1 μM | 9.86 ±0.1**** | 9.2 ±4.5* | 145.4 ±3.4**** | 139.6 ±6.4**** | 135.2 ±5.5 | 5.83 ±0.2 |
|  | 10 μM | 9.24 ±0.1**** | 24.9 ±4.3**** | 149.9 ±2.7**** | 142.3 ±4.9**** | 125.1 ±4.9 |  |
| ^a^Negative logarithm of NECA concentration required to produce a half-maximal response in the absence (NECA only or IB-MECA only) or presence of compound  ^b^Minimum cAMP accumulation of NECA as % forskolin response; the lower plateau of the fitted sigmoidal dose response curve  ^c^The upper plateau of the fitted sigmoidal dose response curve corresponding to 100% of the forskolin response  ^d^The cAMP accumulation when stimulated with compound at the indicated concentration and forskolin stimulation only  ^e^The difference between E_min_ and basal signalling  ^f^Value reported to determine inverse agonism: Negative logarithm of compound concentration required to produce a half-maximal response  Statistical significance (**, p< 0.05; **, p<0.01; ***, p<0.001; ****, p<0.0001*) compared to NECA/IB-MECA only stimulation was determined by one-way ANOVA with Dunnett’s post-test. | | | | | | | |

**Supplementary Figure 6. Antagonism of the HEMADO induced cAMP inhibition at the A_3_R of A) K17 and B) K18.** A_3_R stably expressing Flp-In CHO cells (2000 cells/well) were exposed to forskolin 10 μM, HEMADO and DMSO/test compound at the indicated concentration for 30 min and cAMP accumulation detected. All values are mean ± SEM expressed as percentage forskolin inhibition (10 μM) relative to HEMADO. *n* = 3 independent experimental repeats, conducted in duplicate.

| **Supplementary Table 6. cAMP accumulation as measured in Flp-In-CHO cells stably expressing A_3_R following stimulation with 10 μM** **forskolin, HEMADO and varying concentrations of test compound** | | | | | | |
| --- | --- | --- | --- | --- | --- | --- |
| **A_3_R CHO-K1** | | | | | | |
|  |  | **pIC_50_ ^a^** | **E_min_ ^b^** | **Basal ^c^** | **True Basal ^d^** | **Span ^e^** |
| **HEMADO only** |  | 10.54 ±0.2 | -3.48 ±2.7 | 103.8 ±4.1 | 100.1 ±1.9 | 107.3 ±4.7 |
| **K17** | 0.1 μM | 10.45 ±0.1 | -1.31 ±2.8 | 114.3 ±4.0 | 107.6 ±3.6 | 115.6 ±4.6 |
|  | 1 μM | 9.89 ±0.1 | 2.71 ±2.9 | 134.0 ±3.3 | 126.4 ±4.1 | 131.3 ±4.3 |
|  | 10 μM | 8.99 ±0.1 | 12.16 ±4.1 | 143.6 ±3.4 | 133.3 ±9.3 | 131.5 ±5.2 |
| **K18** | 0.1 μM | 10.04 ±0.1 | 1.20 ±2.9 | 121.2 ±3.5 | 109.1 ±2.2 | 120.0 ±4.4 |
|  | 1 μM | 9.34 ±0.1 | 9.52 ±3.9 | 139.5 ±2.8 | 130.1 ±5.3 | 130.0 ±3.9 |
|  | 10 μM | 8.12 ±0.1 | 19.6 ±4.6 | 141.4 ±2.8 | 130.2 ±9.3 | 121.8 ±5.3 |
| ^a^Negative logarithm of HEMADO concentration required to produce a half-maximal response in the absence (HEMADO only) or presence of compound  ^b^Minimum cAMP accumulation of HEMADO as % 10 μM forskolin response; the lower plateau of the fitted sigmoidal dose response curve  ^c^The upper plateau of the fitted sigmoidal dose response curve corresponding to 100% of the 10 μM forskolin response  ^d^The cAMP accumulation when stimulated with compound at the indicated concentration and 10 μM forskolin stimulation only  ^e^The difference between E_min_ and basal signalling | | | | | | |

**Supplementary Figure 7. A_3_R shows constitutive activity.** cAMP accumulation following a 30-minute stimulation with forskolin (5 μM and 10 μM) in WT A_3_R expressing Flp-In-CHO cells was reduced compared to control (Flp-In-CHO cells). Statistical significance (****, p<0.001*) compared to control was determined by Student’s t-test.

**
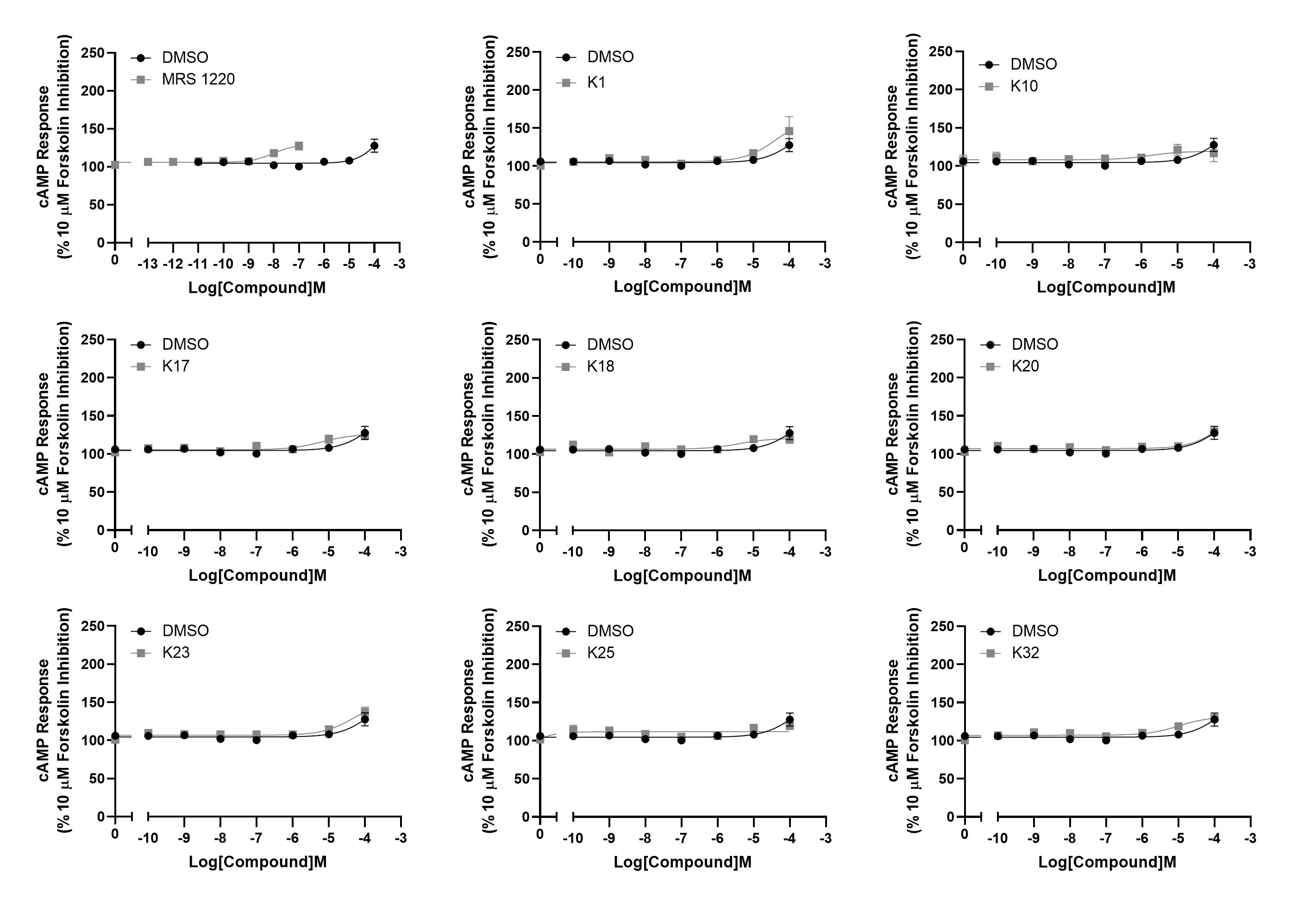
**

**Supplementary Figure 8. Determining A_3_R-dependent inverse agonism of compounds.** WT Flp-In-CHO cells were exposed to forskolin (10 μM) and increasing concentrations of agonist (NECA/IB-MECA), test compound or DMSO control for 30 min and cAMP accumulation detected. Representative curves are shown as mean ± SEM expressed as percentage forskolin (10 μM).

**Supplementary Figure 9. A_3_R stimulated pERK activity is entirely G_i/o_ mediated.** pERK was detected in Flp-In-CHO cells stably expressing A_3_R (2000 cells/well) stimulated for 5 minutes with NECA or IB-MECA with or without Pertussis toxin (PTX) treatment (16 hours at 100 ng/mL). All values are mean ± SEM expressed as % 1μM PMA response where *n* =3 for none-PTX treated and *n* = 1 for PTX treated, conducted in duplicate.


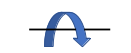

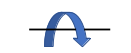

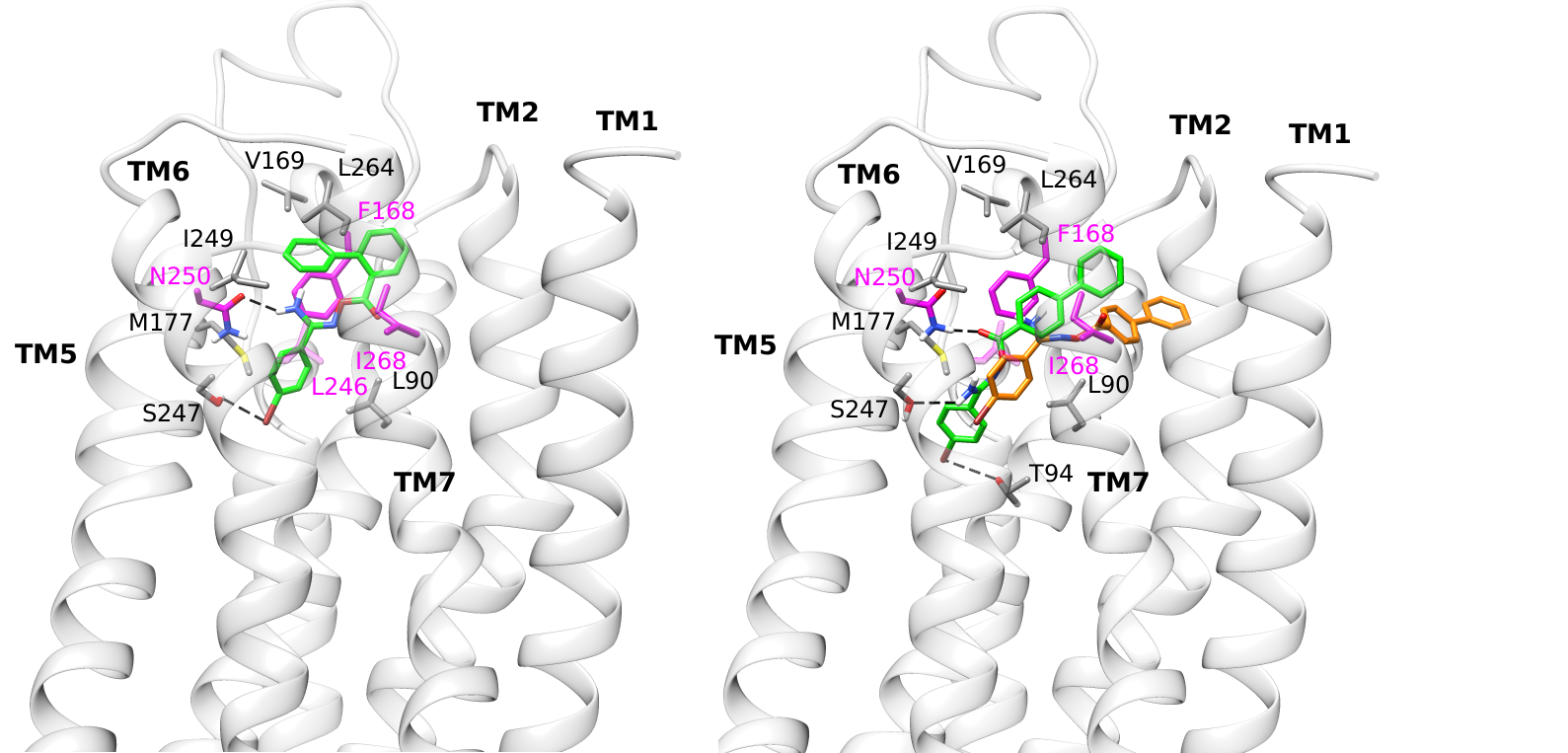

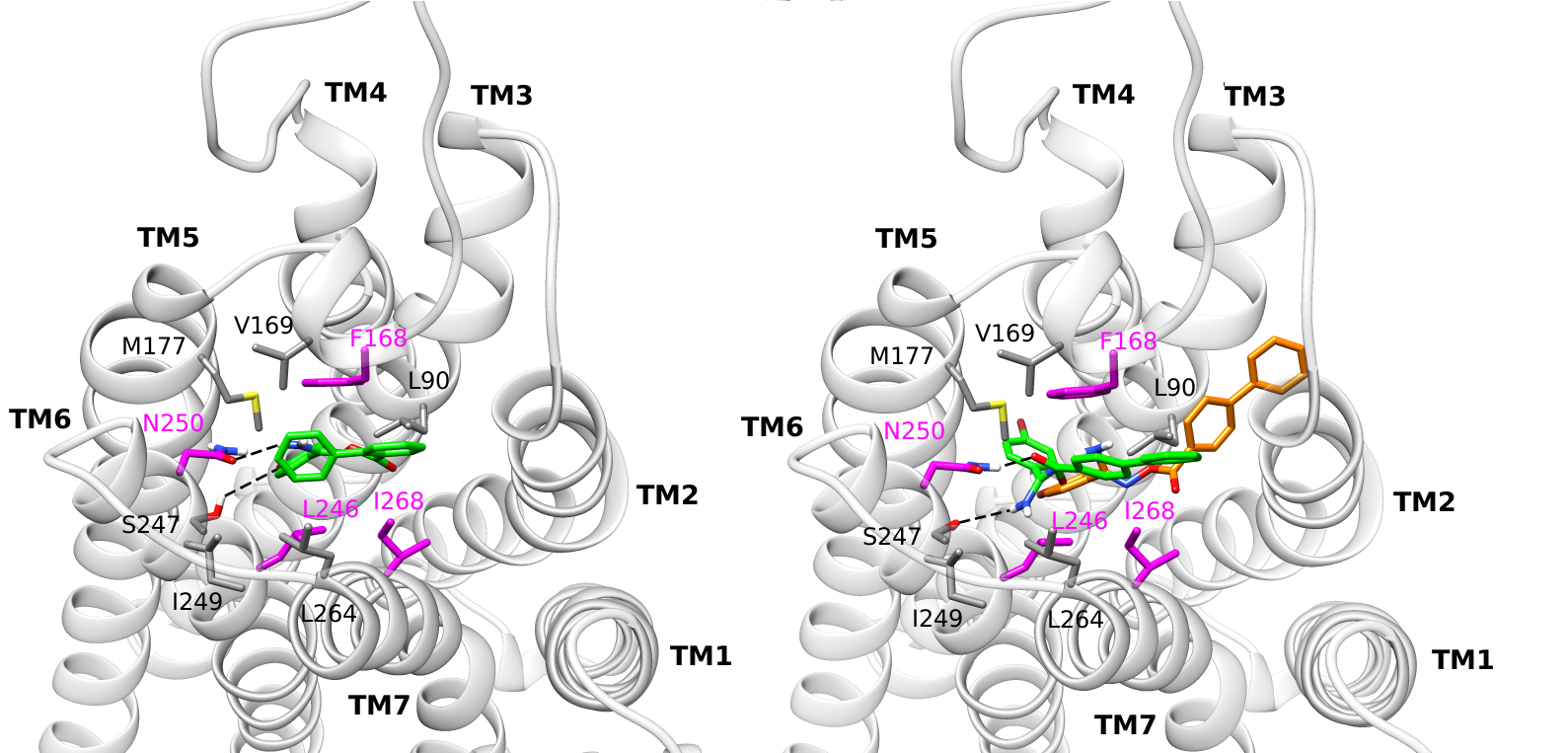


**(C) (D)**

**(A) (B)**

**Supplementary Figure 10. Average structure of WT A_3_R in complex with K26 and K36 from MD simulations with Amber14ff.** Side **(A)**, top **(C)** view of the average structure of K26 which binds A_3_R (*K*_d_ =5.07 μΜ) but has not antagonistic activity and, side **(B)**, top **(D**) view of the average structure of K36 which did not bind A_3_R inside the orthosteric binding area from 100 MD simulations. Side chains of critical residues for binding resulted from the MD simulations are shown in sticks. Residues L90^3.32^, V169^5.30^, M177^5.40^, I249^6.54^, L264^7.34^, in which carbon atoms are shown in grey, were confirmed experimentally; in residues F168^5.29^, L246^6.51^, I268^7.39^ and N250^6.55^ carbon atoms are shown in magenta; nitrogen, oxygen and sulfur atoms are shown in blue, red and yellow respectively. For K36 the conformation adopted after 100 ns, which loses binding interactions with the receptor area, is indicated with orange colour for carbons.

**Supplementary Figure 11. pA_2_ values obtained through Schild analysis are agonist independent.** Flp-In-CHO cells (2000 cells/well) stably expressing WT or L90A^3.32^  A_3_R were exposed to forskolin 10 μM, agonist (NECA or IB-MECA) and K18 at varying concentrations for 30 min and cAMP accumulation detected. IC_50_ values determined through fitting three-parameter logistic equation to concentration response data were used to conduct Schild analysis.

**Supplementary Figure 12. Kinetic measurements of CA200645 binding to Nluc-A_3_R.** HEK 293 cells stably expressing Nluc-A_3_R where stimulated with the fluorescent ligand CA200645 at the indicated concentration. BRET between Nluc and the CA200645 was measured every 5 seconds for 30 minutes at room temperature. Determined kinetic parameters for CA200645 at Nluc-A_3_R were K_on_ = 2.86 ± 0.89 x 10^7^ M^-1^ and K_off_ = 0.4397 ± 0.014 min^-1^ with a resulting K_D_ of 17.92 ± 4.45 nM. Data were baseline corrected and shown here as representative of five independent experiments, conducted in duplicate.

**Supplementary Figure 13. Intrinsic clearance of K18.** The metabolic stability of K18 (0.1 μM) was studied using human liver microsomes (0.1 mg/mL) to derive the metabolic half-life (*t_1/2_*) from the slope (k). The *t_1/2_* of K18 was determined as 24 minutes using the equation: *t_1/2_* = - In(2)/k. Intrinsic clearance (CL_int_) was calculated as 287.2 μl/min/mg. Each data point represents the mean ± SEM of a single test conducted in duplicate.

**Appendix I: Adapting the kinetics of competitive binding equation for rapidly-dissociating unlabelled ligands**

The Motulsky and Mahan equation is used to measure the association and dissociation rate constant of unlabelled compounds, in competition with labelled ligand for binding to a receptor^1^. When the unlabelled competitor dissociates rapidly, relative to the early time points of the assay, the fitted parameters can indicate a failure of the fit to provide realistic or sufficiently precise estimates of the model parameters. Here an equation is derived for rapidly-dissociating compounds that provides an estimate of the equilibrium binding affinity rather than the binding rate constants of the unlabelled competitor. It is assumed the competitor is at equilibrium throughout the time course of the binding assay. This is represented by the following scheme, where *K*_i_ is the equilibrium dissociation constant of the competitor:


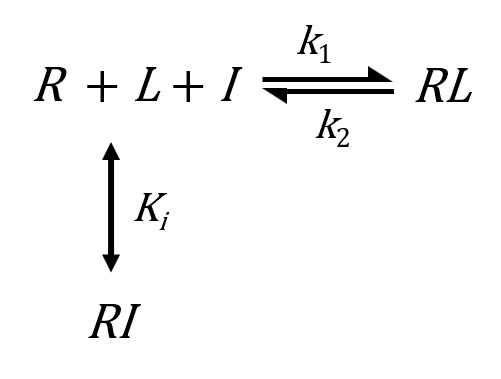


*R* is receptor, *L* is labelled ligand, *I* is unlabelled competitor, *k*_1_ is the association rate constant and *k*_2_ the dissociation rate constant of the labelled ligand. The assumptions underlying the original kinetics of competitive binding equation apply^1^: Only a small fraction (< 10%) of total tracer and compound concentration is bound by receptor (“Zone A”); receptor is exposed simultaneously to both ligands; receptor-ligand binding conforms to a single-site, single-step non-cooperative mass-action mechanism; and unlabelled ligand competitively inhibits tracer binding.

The goal is an equation that describes binding of labelled ligand to receptor over time in the presence of unlabelled competitor. We start with the differential equation for [*RL*]:

$$\frac{d[RL]}{dt}=\left[ R \right]\left[ L \right]k_{1}-[RL]k_{2}$$

Next, *R* is substituted by employing the conservation of mass equation:

$$N=\left[ R \right]+[RI]+\left[ RL \right]$$

where *N* is the total concentration of receptor. Solving for [*R*] gives,

$$\left[ R \right]=N-\left[ RI \right]-\left[ RL \right]$$

This expression is now substituted into the differential equation for [*RL*]:

$$\frac{d[RL]}{dt}=N\left[ L \right]k_{1}-\left[ RI \right]\left[ L \right]k_{1}-[RL]\left( \left[ L \right]k_{1}+k_{2} \right)$$

We now substitute [*RI*] with an expression in terms of [*RL*], as follows: Since *I* is at equilibrium with the receptor, the ratio of *R* to *RI* is constant, and can be represented by the following expression:

$$\frac{\left[ RI \right]}{\left[ R \right]+[RI]}=\frac{[I]}{K_{I}+[I]}=\rho_{I}$$

where the constant $\rho_{I}$ is fractional occupancy by *I* of accessible receptors, i.e. those not bound by labelled ligand. Next, this equation is rearranged as follows:

$$\left[ RI \right]=\rho_{I}\left( \left[ R \right]+[RI] \right)$$

The bracketed term on the right-hand side, $\left[ R \right]+[RI]$ can be re-written in terms of [*RL*] and the constant *N*, using the conservation of mass equation for the receptor:

$$\left[ R \right]+\left[ RI \right]=N-\left[ RL \right]$$

$\left[ R \right]+[RI]$ is now substituted with $N-\left[ RL \right]$:

$$\left[ RI \right]=\rho_{I}\left( N-\left[ RL \right] \right)$$

This is the desired expression: [*RI*] is expressed in terms of [*RL*]. This expression is now substituted into the differential equation for [*RL*], which gives, after rearranging,

$$\frac{d[RL]}{dt}=N\left[ L \right]k_{1}\left( 1-\rho_{I} \right)-\left[ RL \right]\left\{ \left[ L \right]k_{1}\left( 1-\rho_{I} \right)+k_{2} \right\}$$

This equation is now integrated to obtain the [*RL*] *vs t* equation that can be used to fit experimental data:

$${[RL]}_{t}=\frac{N\left[ L \right]k_{1}\left( 1-\rho_{I} \right)}{k_{obs,+I}}\left( 1-e^{-k_{obs,+I}t} \right)$$

where

$$k_{obs,+I}=\left[ L \right]k_{1}\left( 1-\rho_{I} \right)+k_{2}$$

and

$$\rho_{I}=\frac{[I]}{K_{I}+[I]}$$

**Appendix II: Supporting information for computational biochemistry**

**Preparation of the structures**

Structures of the compounds K5, K17, K18 or MRS 1220 were prepared using Maestro (Version 10.5; Schrodinger, Inc.: New York, NY, 2015) and minimized as in a previous paper^2^. The inactive state homologue of A_3_R WT was taken from Adenosiland web-service^3^ . The BLAST algorithm estimated the human A_1_R^4^ (PDB ID 5UEN) as the most appropriate template for human A_3_R model having the most similar sequence. The rat A_3_R was generated using the protein structure homology model server SwissModel^5^. We also applied MODELLER 9.18^6,7^ which selected ten PDB structures with the highest sequential similarity as templates for homology modeling of rat A_3_R. Twenty homology models were generated and the model with the lowest DOPE (Discrete Optimized Protein Energy) value was selected. The two resulting rat A_3_R models created by Swiss Model and MODELLER 9.18 were compared to each other using the Protein Structure Alignment Tool of Desmond Maestro 2018-1 and were found to be very similar (Desmond Molecular Dynamics System, version 3.0; D.E. Shaw Res. New York, 2011; Maest. Interoperability Tools, 3.1; Schrodinger Res. New York, 2012).

The protein models were optimized as previously published using the Protein Preparation Wizard implementation in Schrodinger suite (Protein Prep. Wizard 2015-2; Epik version 2.4, Schrödinger, LLC, New York, NY, 2015; Impact version 5.9, Schrödinger, LLC, New York, NY, 2015; Prime version 3.2, Schrödinger, LLC, New York, NY, 2015). The ZM241385-inactive A_2A_R protein complex from 3EML^8^ was superimposed to human or rat A_3_R WT and the A_2A_R protein was removed resulting in ZM241385-A_3_R complex which was used as a template for docking of K5, K17, K18, K25 or MRS 1220 using GoldScore and ChemPLP scoring functions^9,10^ and the GOLD (Version 5.2, Cambridge Crystallogr. Data Cent. Cambridge, U.K., 2015)^11^ as previously described^2^. The top high-scoring poses for K5, K17, K18, K25 or MRS 1220 in complex with A_3_R using GoldScore were better and were kept. These complexes were embedded in POPE bilayers using the System Builder utility of Desmond (Desmond Molecular Dynamics System, version 3.0; D.E. Shaw Res. New York, 2011; Maest. Interoperability Tools, 3.1; Schrodinger Res. New York, 2012). Complex and lipid systems were solvated using the TIP3P water model^12^. Na^+^ and Cl^-^ ions were placed in the water phase to neutralize the systems and to reach the experimental salt concentration of 0.150 M NaCl. A 10 Å-from the solute atoms-buffered orthorhombic system with periodic boundary conditions was constructed for all complexes.

**MD simulations**

Each ligand-A_3_R complex in the bilayer was processed by the LEaP module in AmberTools14 under the AMBER14 software package^13^*.* Amber ff14SB force field parameters^14^ was applied to the protein, lipid14 to the lipids^15^, GAFF to the ligands^16^ and TIP3P^12^ to the water molecules for the calculation of bonded, vdW parameters and electrostatic interactions. Atomic charges were computed according to the RESP procedure using Gaussian03^17^ and *antechamber* of AmberTools14. MD simulations in explicit solvent were performed using PMEMD^13^. MD simulation protocol consists of five stages: a) Minimization, b) Heating, c) Adjustment of density, d) Equilibration and e) Production. The systems were minimized by 2500 steps of steepest descent to remove bad contacts and 7500 steps of conjugated gradient minimization in the presence of a harmonic restraint with a force constant of 5 kcal mol^-1^ Å^-2^ on all atoms of protein and ligand and non-bonded cut-off of 8.0 Å. The next stage in MD simulation protocol is to allow the system to heat up from 0 K to 310 K. Langevin thermostat (dynamics)^18^ as implemented in Amber14^13^ was used for temperature control employing a Langevin collision frequency of 2.0 ps^‑1^. The system in two consecutive steps to 310 K in the presence of a harmonic restraint with a force constant of 10 kcal mol^-1^ Å^-2^ on all membrane, protein, and ligand atoms. In the first step, systems were heated to 100 K in a NVT of 50 ps length where the adjustment of the density was realized using the Berendsen barostat^19^ with a 2 ps coupling time. In the second step, the temperature was raised to 310 K in a NPT*γ* (with *γ*= 10 dyn cm^‑1^) simulation of 500 ps length. Subsequently, the systems were equilibrated without restraints in a NPTγ simulation of 1 ns length with *T*= 310 K and γ = 10 dyn cm^‑1^. The equilibration phase was followed by production simulation for 100 ns with system-specific lengths using the same protocol as in the final equilibration step. In the NPTγ simulations semiisotropic pressure scaling to *p*= 1 bar was applied using a pressure relaxation time of 1.0 ps. For the treatment of long-range electrostatic interactions, the Particle-mesh Ewald summation method^20,21^ was used, and short-range non-bonding interactions were truncated with an 8 Å cutoff. Bonds involving hydrogen atoms were constrained by the SHAKE algorithm^22^, and a time step of 2 fs was used for the integration of the equations of motion. Snapshots recorded every 20 ps during the production Properties and dynamics of the protein and ligand systems as well as of the membrane were analyzed with the *ptraj* and *cpptraj* modules of AmberTools12^12^.

**MM-PBSA calculations**

For this, structural ensembles were extracted in intervals of 50 ps from the last 50 ns of the production simulations for each complex. Prior to the calculations all water molecules, ions, and lipids were removed, and the structures were positioned such that the geometric centre of each complex was located at the coordinate origin. The polar part of the solvation free energy was determined by calculations using Poisson-Boltzmann (PB) calculations^23^. In these calculations, a dielectric constant of *ε*_solute_ = 1 was assigned to the binding area and *ε*_solute_ = 80 for water. Using an implicit solvent representation for the calculation of the effective binding energy is an approximation to reduce the computational cost of the calculations. The binding free energy for each complex was calculated using equation (1)

Δ*G*_eff_ = Δ*E*_MM_ + Δ*G*_sol_ (1)

In equation (1) Δ*G*_eff_ is the binding free energy for each calculated complex neglecting the effect of entropic contributions or assuming to be similar for the complexes studied. Δ*E*_MM_ defines the interaction energy between the complex, the protein and the ligand as calculated by molecular mechanics in the gas phase. Δ*G*_sol_ is the desolvation free energy for transferring the ligand from water in the binding area calculated using the PBSA model. The terms for each complex Δ*E*_MM_ and Δ*G*_sol_ are calculated using equations (2) and (3)

Δ*E*_MM_ = Δ*E*_elec_ + Δ*E*_vdW_ (2)

Δ*G*_sol_ = Δ*G*_P_ + Δ*G*_NP_ (3)

In equation (2) Δ*E*_elec_ and Δ*E*_vdW_ are the electrostatic and the vdW interaction energies, respectively. In equation (3) Δ*G*_P_ is the electrostatic or polar contribution to free energy of solvation and the term Δ*G*_NP_ is the non-polar or hydrophobic contribution to solvation free energy. Molecular mechanics energies and the non-polar contribution to the solvation free energy were calculated with the *mmpbsa.pl* module^24^ of Amber14^12^ (Case *et al.,* 2014).

**REFERENCES**

1. Motulsky, H. J. (n.d.). Interpreting the extra sum-of squares F test. Retrieved May 30, 2019, from Motulsky, H. J., & Mahan, L. C. (1984). The Kinetics of Competitive Radioligand Binding Predicted by the Law of Mass-Action. *Molecular Pharmacology*, ***25***(1), 1–9.

2. Lagarias, P., Vrontaki, E., Lambrinidis, G., Stamatis, D., Convertino, M., Ortore, G., et al. (2018). Discovery of Novel Adenosine Receptor Antagonists through a Combined Structure- and Ligand-Based Approach Followed by Molecular Dynamics Investigation of Ligand Binding Mode. *Journal of Chemical Information and Modeling*, ***58***(4), 794–815. DOI: 10.1021/acs.jcim.7b00455

3. Floris, M., Sabbadin, D., Medda, R., Bulfone, A., & Moro, S. (2012). Adenosiland: walking through adenosine receptors landscape. *European Journal of Medicinal Chemistry*, ***58***, 248–257. DOI: 10.1016/j.ejmech.2012.10.022

4. Glukhova, A., Thal, D. M., Nguyen, A. T., Vecchio, E. A., Jörg, M., Scammells, P. J., et al. (2017). Structure of the Adenosine A1 Receptor Reveals the Basis for Subtype Selectivity. *Cell*, ***168***(5), 867–877.e13. http://doi.org/10.1016/j.cell.2017.01.042

5. Waterhouse, A., Bertoni, M., Bienert, S., Studer, G., Tauriello, G., Gumienny, R., et al. (2018). SWISS-MODEL: homology modelling of protein structures and complexes. *Nucleic Acids Research*, ***46***(W1), W296–W303.

6. Sali, A., & Blundell, T. L. (1993). Comparative protein modelling by satisfaction of spatial restraints. *Journal of Molecular Biology*, ***234***(3), 779–815. http://doi.org/10.1006/jmbi.1993.1626

7. Eswar, N., John, B., Mirkovic, N., Fiser, A., Ilyin, V. A., Pieper, U., et al. (2003). Tools for comparative protein structure modeling and analysis. *Nucleic Acids Research*, ***31***(13), 3375–3380. http://doi.org/10.1093/nar/gkg543

8. Jaakola, V.-P., Griffith, M. T., Hanson, M. A., Cherezov, V., Chien, E. Y. T., Lane, J. R., et al. (2008). The 2.6 angstrom crystal structure of a human A2A adenosine receptor bound to an antagonist. *Science (New York, N.Y.)*, ***322***(5905), 1211–1217. DOI: 10.1126/science.1164772

9. Jones, G., Willett, P., Glen, R. C., Leach, A. R., & Taylor, R. (1997). Development and validation of a genetic algorithm for flexible docking. *Journal of Molecular Biology*, ***267***(3), 727–748. DOI: 10.1006/jmbi.1996.0897

10. Eldridge, M. D., Murray, C. W., Auton, T. R., Paolini, G. V., & Mee, R. P. (1997). Empirical scoring functions: I. The development of a fast empirical scoring function to estimate the binding affinity of ligands in receptor complexes. *Journal of Computer-Aided Molecular Design*, ***11***(5), 425–445.

11. Verdonk, M. L., Chessari, G., Cole, J. C., Hartshorn, M. J., Murray, C. W., Nissink, J. W. M., et al. (2005). Modeling water molecules in protein-ligand docking using GOLD. *Journal of Medicinal Chemistry*, ***48***(20), 6504–6515. DOI: 10.1021/jm050543p

12. Jorgensen, W. L., Chandrasekhar, J., Madura, J. D., Impey, R. W., & Klein, M. L. (1983a). Comparison of Simple Potential Functions for Simulating Liquid Water. *Journal of Chemical Physics*, ***79***(2), 926–935. DOI: 10.1063/1.445869

13. Case, D. A., Babin, V., Berryman, J. T., Betz, R. M., Cai, Q., Cerutti, D. S., & Kollman, P. A. (2014). AMBER 14. University of California, San Francisco.

14. Maier, J. A., Martinez, C., Kasavajhala, K., Wickstrom, L., Hauser, K. E., & Simmerling, C. (2015). ff14SB: Improving the Accuracy of Protein Side Chain and Backbone Parameters from ff99SB. *Journal of Chemical Theory and Computation*, ***11***(8), 3696–3713. DOI: 10.1021/acs.jctc.5b00255

15. Dickson, C. J., Madej, B. D., Skjevik, A. A., Betz, R. M., Teigen, K., Gould, I. R., & Walker, R. C. (2014). Lipid14: The Amber Lipid Force Field. *Journal of Chemical Theory and Computation*, ***10***(2), 865–879. DOI: 10.1021/ct4010307

16. Wang, J. M., Cieplak, P., & Kollman, P. A. (2000). How well does a restrained electrostatic potential (RESP) model perform in calculating conformational energies of organic and biological molecules? *Journal of Computational Chemistry*, ***21***(12), 1049–1074. DOI: 10.1002/1096-987X(200009)21:12<1049::AID-JCC3>3.3.CO;2-6

17. Frisch, M. J., Trucks, G. W., Schlegel, H. B., Scuseria, G. E., Rob, M. A., & Pople, J. A. (2003). *Gaussian 03*. Wallingford, CT.

18. Izaguirre, J. A., Catarello, D. P., Wozniak, J. M., & Skeel, R. D. (2001). Langevin stabilization of molecular dynamics. *Journal of Chemical Physics*, ***114***(5), 2090. DOI: 10.1063/1.1332996

19. , H. J. C., Postma, J. P. M., van Gunsteren, W. F., DiNola, A., & Haak, J. R. (1984). Molecular dynamics with coupling to an external bath. *Journal of Chemical Physics*, ***81***(8), 3684. DOI: 10.1063/1.448118

20. Darden, T., York, D., & Pedersen, L. (1993). Particle mesh Ewald: An N⋅log(N) method for Ewald sums in large systems. *Journal of Chemical Physics*, ***98***(12), 10089. DOI: 10.1063/1.464397

21. Essmann, U., Lalith Perera, Berkowitz, M. L., Darden, T., Lee, H., & Pedersen, L. G. (1995). A smooth particle mesh Ewald method. *Journal of Chemical Physics*, ***103***(19), 8577. DOI: 10.1063/1.470117

22. Ryckaert, J.-P., Ciccotti, G., & Berendsen, H. J. C. (1977). Numerical integration of the Cartesian Equations of Motion of a System with Constraints: Molecular Dynamics of n- Alkanes. *Journal of Computational Physics*, ***23***(3), 327–341.

23. Homeyer, N., & Gohlke, H. (2013). FEW: A workflow tool for free energy calculations of ligand binding. *Journal of Computational Chemistry*, ***34***(11), 965–973. DOI: 10.1002/jcc.23218

24. Miller, B. R. I., McGee, T. D. J., Swails, J. M., Homeyer, N., Gohlke, H., & Roitberg, A. E. (2012). MMPBSA.py: An Efficient Program for End-State Free Energy Calculations. *Journal of Chemical Theory and Computation*, ***8***(9), 3314–3321. DOI: 10.1021/ct300418h
